# Synthesis and Pharmacological Evaluation of Novel Pleuromutilin Derivatives with Substituted Benzimidazole Moieties

**DOI:** 10.3390/molecules21111488

**Published:** 2016-11-08

**Authors:** Xin Ai, Xiuying Pu, Yunpeng Yi, Yu Liu, Shuijin Xu, Jianping Liang, Ruofeng Shang

**Affiliations:** 1 Key Laboratory of New Animal Drug Project of Gansu Province, Key Laboratory of Veterinary Pharmaceutical Development, Ministry of Agriculture, Lanzhou Institute of Husbandry and Pharmaceutical Sciences of CAAS, 335 Jiangouyan, Lanzhou 730050, China; ai_xin2015@163.com (X.A.); yiyunpeng88@163.com (Y.Y.); yangguang8684@163.com (Y.L.); liangjp1963@163.com (J.L.); 2 College of Life Science and Engineering, Lanzhou University of Technology, 287 Langongping Road, Lanzhou 730050, China; puhl02@163.com; 3 Yancheng Shunbao Chemical Co., Ltd., Yancheng 224555, China; xu@youhuapharm.com

**Keywords:** pleuromutilin derivatives, synthesis, antibacterial activity, inhibition, CYP3A4

## Abstract

A series of novel pleuromutilin derivatives with substituted benzimidazole moieties were designed and synthesized from pleuromutilin and 5-amino-2-mercaptobenzimidazole through sequential reactions. All the newly synthesized compounds were characterized by IR, NMR, and HRMS. Each of the derivatives was evaluated in vitro for their antibacterial activity against *Escherichia coli* (*E. coli*) and five Gram (+) inoculums. 14-*O*-((5-amino-benzimidazole-2-yl) thioacetyl) mutilin (**3**) was the most active compound and showed highest antibacterial activities. Furthermore, we evaluated the inhibition activities of compound **3** on short-term *S. aureus* and MRSA growth and cytochrome P450 (CYP). The bioassay results indicate that compound **3** could be considered potential antibacterial agents but with intermediate inhibition of CYP3A4.

## 1. Introduction

The emergence of multidrug-resistant bacteria has led to the reduction or loss of the curative effects of many available drugs and remains a global human threat with the potential for catastrophic consequences in the future [1]. Infections caused by drug-resistant Gram-positive organisms, such as methicillin-resistant *Staphylococcus aureus* (MRSA) and vancomycin-resistant *enterococci* (VRE), remain a critical issue because of not only the morbidity and mortality caused by these infections but also the associated economic cost [2]. To solve the drastic increase of pathogenic bacterial resistance, especially multiresistant bacteria, there is a pressing need to develop novel antibiotics against these dreadful pathogens.

**Figure 1:**
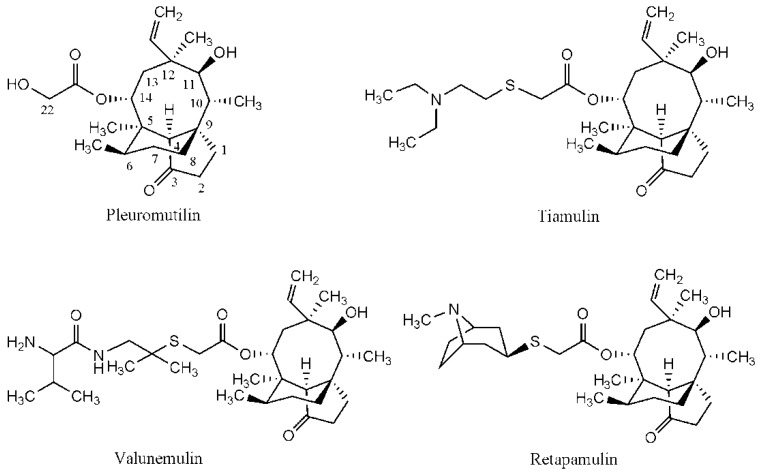
Structural formulas of pleuromutilin, tiamulin, valnemulin, and retapamulin.

The natural compound pleuromutilin (Figure 1) was first discovered and isolated in a crystalline form from cultures of two species of basidiomycetes, *Pleurotus mutilus* and *P. passeckerianus* in 1951 [3]. This compound has a modest antibacterial activity [4]. However, modification of the C-14 position of pleuromutilin may improve its biological activity and thus has led to three drugs: tiamulin, valnemulin, and retapamulin (Figure 1) [5,6,7].

Pleuromutilin derivatives selectively inhibited bacterial protein synthesis by binding to the ribosomes at the acceptor (A) and donor (P) site [8,9]. Further studies demonstrated that the interactions of tricyclic core of the tiamulin are mediated through hydrophobic interactions and hydrogen bond, which are formed mainly by the nucleotides of domain V [10,11]. The C-11 hydroxyl groups and the C-21 keto groups of pleuromutilin derivatives were located in a position suitable for hydrogen bonding to G-2505 phosphate and G-2061, respectively. Meanwhile, C-14 side chains pointed toward the P-site and minor hydrophobic contacts were formed with ribosomal components [11].

Ling [12] and the present authors proposed that heterocyclic rings bearing polar groups at the C14 side chain of pleuromutilin derivatives improved their antibacterial activity [13,14,15]. Two classes of pleuromutilin derivatives with 1,3,4-thiadiazole and pyrimidine ring were reported and selected as a few compounds with excellent in vitro antibacterial activity against both sensitive and resistant Gram-positive bacterial strains. In this work, we describe the design, synthesis, and antibacterial studies for a novel pleuromutilin derivatives with a substituted benzimidazole moiety. This new series bears the fused heterocyclic ring at the C14 side chain of pleuromutilin derivatives instead of a single heterocyclic structure and thus are distinct from previous reported compounds.

## 2. Results and Discussion

### 2.1. Chemistry

**Scheme 1:**
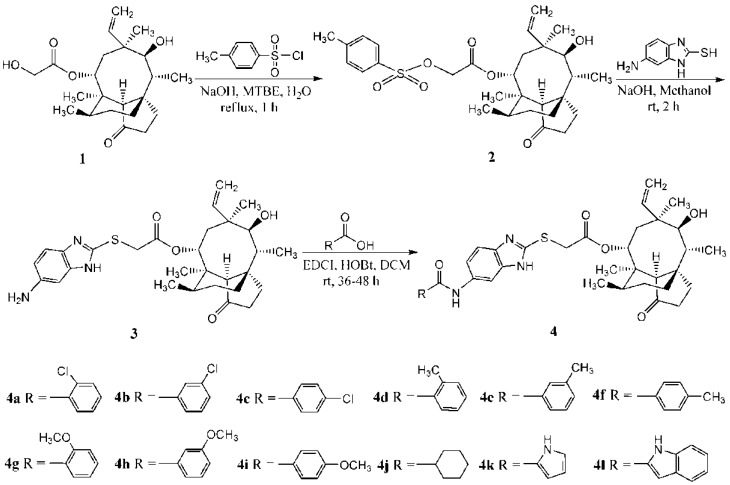
General synthetic scheme for the pleuromutilin derivatives.

The general synthetic route to building the pleuromutilin derivatives is illustrated in Scheme 1. The lead compound 14-*O*-((5-amino-benzimidazole-2-yl) thioacetyl) mutilin (**3**) was prepared by nucleophilic substitution of 22-*O*-tosylpleuromutilin (**2**) with 5-amino-2-mercaptobenzimidazole under basic conditions with a 66% yield. Compounds **4a**–**l** were directly obtained with a 51%–94% yield via condensation reactions between the amino group of compound **3** and the carboxyl group of carboxylic acids. The reactions were performed at room temperature in the presence of 1-ethyl-3-(3-dimethyllaminopropyl) carbodiimide hydrochloride (EDCI) and 1-hydroxybenzotriazole (HOBt), which was used to suppress racemization and improve the efficiency of the amide synthesis [13]. The synthesis and the IR, ^1^H-NMR, and ^13^C-NMR spectra of all the new compounds are reported in the Supplementary data.

### 2.2. Antibacterial Activity

The synthesized pleuromutilin derivatives **3** and **4a**–**l**, with tiamulin fumarate used a as reference drug, were screened for their in vitro antibacterial activity against *Escherichia coli* (*E. coli*), *Staphylococcus aureus* (*S. aureus*), *Methicillin-resistant Staphylococcus aureus* (MRSA), *Staphylococcus vitulinus* (*S. vitulinus*), *Staphylococcus warneri* (*S. warneri*), and *Staphylococcus haemolyticus* (*S. haemolyticus*). The antibacterial activities are reported in Table 1 as the minimum inhibitory concentration (MIC) using the agar dilution method. The MICs of the 13 new pleuromutilin derivatives in vitro against *E. coli* ranged from 10 to 80 μg/mL; against *S. aureus*, MRSA, *S. vitulinus*, *S. warneri*, and *S. haemolyticus* ranged from 0.156 to 40 μg/mL, respectively. It can be observed that compound **3** showed the highest antibacterial activities than the other synthesized compounds and was comparable to the reference drug. Compounds **4j**–**l** exhibited relatively moderate inhibitory activities against *S. aureus*, MRSA, *S. vitulinus*, *S. warneri*, and *S. haemolyticus* and weak activity against *E. coli.* However, the other compounds showed lower antibacterial activities.

The Oxford cup assays were carried out to evaluate the antibacterial activities against the above-mentioned six bacterial strains. The zones of inhibition for two concentrations (320 and 160 μg/mL) of the synthetic compounds and tiamulin fumarate were measured. Data are reported as diameters of growth inhibition (mm), and the results are given in Table 2. The results of Oxford cup assay correspond with that obtained by agar dilution method (MICs) as a whole. Compound **3** showed the best growth inhibition against the pathogens particularly haemolyticus and comparable to tiamulin, whereas compounds **4j**–**l** showed moderate growth inhibition except against *E. coli*.

**Figure 2:**
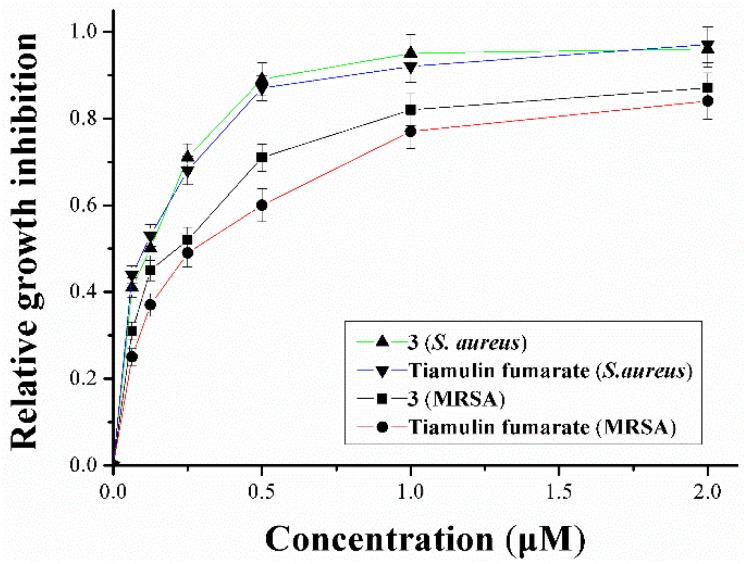
Inhibition of the growth of *S. aureus* and MRSA by compound **3** and tiamulin fumarate. Data points are the mean from at least three independent experiments carried out in duplicate. The standard deviation is shown as error bars.

Compound **3**, being the best antibacterial derivative, was chosen for further evaluation on its antibacterial effect to inhibit short-term *S. aureus* and MRSA growth (Figure 2). This includes the GI_50_ of tiamulin fumarate for *S. aureus* and MRSA. The inhibition study revealed that compound **3** and tiamulin fumarate showed higher inhibition efficacy against *S. aureus* than that against MRSA, respectively. Compared with tiamulin fumarate (GI_50_ was 0.2535 for MRSA), compound **3** displayed higher growth inhibition with a GI_50_ of 0.1748 μM against MRSA. However, there was no significant difference between compound **3** (GI_50_ was 0.0987 for *S. aureus*) and tiamulin fumarate (GI_50_ was 0.0983 for *S. aureus*) when they inhibited *S. aureus* growth.

### 2.3. Inhibitory Effects on Cytochrome P450

**Figure 3:**
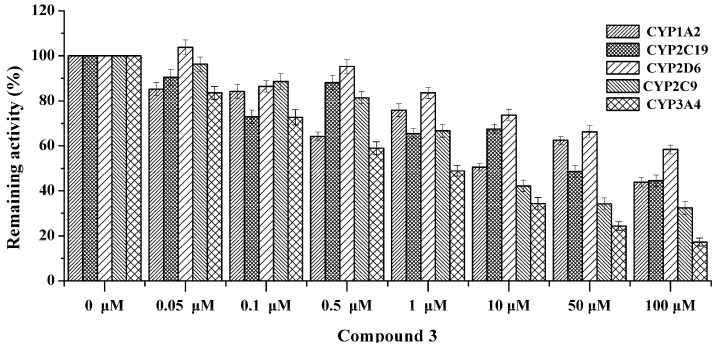
Inhibitory effects of compound **3** on CYP-catalyzed reactions. Each data point represents the mean of triplicate experiments and the standard deviation is shown as error bars.

The cytochrome P450 (CYP) proteins form a large family of heme proteins that catalyze many reactions involved xenobiotic metabolism and biosynthesis of cholesterol, steroids, and other lipid components. Some chemicals could affect the disposition of conventional pharmaceuticals through the inhibition of CYP450 enzymes. The in vitro inhibitory effects of compound **3** on the activities of five common major human CYP 450 were evaluated, as some pleuromutilin derivatives showed potent inhibition of CYP3A4, especially azamulin, a synthetic azole pleuromutilin [16]. The selective probe substrate concentrations were prepared near to its respective *Km* value, and the compound **3** concentration range is from 0.05 to 100 mM. The CYP 450 inhibition was analyzed by determining IC_50_ values, and the results are summarized in Table 3 and Figure 3.

Compound **3** showed intermediate inhibition of CYP3A4 and CYP2C9, with IC_50_ values of 1.69 and 10.70 μM, respectively. In contrast, compound **3** displayed low to no significant inhibitory effects on CYP1A2 (IC_50_ > 50), CYP2C19 (IC_50_ > 50), and CYP2D6 (IC_50_ > 100). It was reported that the selective inhibition for CYP3A4 may be primarily a function of the pleuromutilin portion of the molecule [16,18]. However, side chain structures of pleuromutilin derivatives have some influence on CYP3A4 inhibition profiles [12]. Our studies show that compound **3** is potent inhibitor of CYP3A4, a member of the CYP450 family with the most common and the most versatile function. Further research on design and synthesis of new pleuromutilin derivatives should be considered to improve CYP450 inhibition.

## 3. Experimental Section

### 3.1. Synthesis

#### 3.1.1. General

Starting materials, reagents, and solvents were of analytical grade and obtained from commercial sources. All reactions were monitored by thin-layer chromatography (TLC) analysis on silica gel GF254 plates and visualized under UV illumination at 254 nm for UV active materials after elution. Further visualization was achieved by staining with a 0.05% KMnO_4_ aqueous solution. All column chromatography purifications were carried out on silica gel (200–300 mesh, Qingdao Haiyang Chemical Co., Ltd., Qingdao, China) through conventional methods. IR spectra were obtained on a NEXUS-670 spectrometer (Nicolet Thermo, Edina, MN, USA) using KBr thin films, and the absorptions are reported in cm^−1^. NMR spectra were recorded in appropriate solvents using a Bruker-400 MHz spectrometer (Bruker BioSpin, Zürich, Switzerland). The chemical shifts (δ) were expressed in parts per million (ppm) relative to tetramethylsilane. The multiplicities of the NMR signals were designated as s (singlet), d (doublet), t (triplet), q (quartet), m (multiplet), br (broad), etc. High-resolution mass spectra (HRMS) were obtained on a Bruker Daltonics APEX II 47e mass spectrometer (Billerica, MA, USA) equipped with an electrospray ion source.

#### 3.1.2. 14-*O*-((5-Amino-benzimidazole-2-yl) thioacetyl) Mutilin (**3**)

A variety of 5-amino-2-mercaptobenzimidazole (1.65 g, 10 mmol) and 10 M NaOH (1.1 mL, 11 mmol) in 50 mL of methanol was stirred for 20 min at room temperature. Compound **2** (0.52 g, 10 mmol) dissolved in 7.5 mL of dichloromethane was added by dropwise. After stirring for 24 h at room temperature, the reaction mixture was evaporated under reduced pressure to dryness. The crude product was extracted three times with a solution of ethyl acetate (60 mL) and water (20 mL). The organic phase was treated with saturated NaHCO_3_ and the target compound **3** was then precipitated without further purification by chromatography to yield 3.47 g (66%). IR (KBr): 3405, 3331, 2935, 1729, 1633, 1445, 1420, 1384, 1359, 1269, 1154, 1118, 1018, 981, 916 cm^−1^; ^1^H-NMR (400 MHz, DMSO) δ 7.33 (s, 1H), 6.74 (s, 1H), 6.62 (d, *J* = 8.5 Hz, 1H), 6.43 (dd, *J* = 17.4 Hz, 11.0, 1H), 5.78 (d, *J* = 8.5 Hz, 1H), 5.21 (dd, *J* = 49.7 Hz, 14.2, 2H), 3.82 (s, 2H), 3.49 (s, 1H), 3.35 (d, *J* = 6.2 Hz, 1H), 2.37–2.28 (m, 1H), 2.27–2.11 (m, 2H), 2.08 (d, *J* = 5.7 Hz, 1H), 2.01 (dd, *J* = 19.6 Hz, 11.0, 1H), 1.77 (d, *J* = 12.0 Hz, 1H), 1.69–1.59 (m, 2H), 1.56–1.21 (m, 9H), 1.17–1.04 (m, 4H), 0.94–0.79 (m, 4H), 0.72 (t, *J* = 7.5 Hz, 3H); ^13^C-NMR (101 MHz, DMSO) δ 216.11, 167.45, 158.40, 137.99, 131.75, 124.81, 121.52, 116.18, 109.05, 73.48, 69.59, 56.99, 52.30, 44.32, 43.37, 42.84, 40.73, 35.60, 34.89, 34.36, 33.36, 29.26, 25.72, 25.29, 23.71, 15.65, 13.69, 10.38; HRMS (ES) calcd [M + H]^+^ for C_29_H_39_N_3_O_4_S 526.2934, found 526.2780.

#### 3.1.3. General Procedure for the Synthesis of Compounds **4a**–**l**

A mixture of the carboxylic acids derivative (2.4 mmol), 1.05 g of compound **3** (2.0 mmol), 0.46 g of 1-ethyl-3-(3-dimethylaminopropyl) carbodiimide hydrochloride (2.4 mmol), and 0.34 g of 1-hydroxybenzotriazole (2.4 mmol) were dissolved in 50 mL of dichloromethane and stirred for 36–48 h at room temperature. The mixture was washed with saturated aqueous NaHCO_3_ and brine, and then dried with Na_2_SO_4_ overnight. The crude residue obtained was purified by silica gel column chromatography (petroleum ether: ethyl acetate 1:1–1:4 *v*/*v*) to afford the desired compounds.

*14-O-((2-Chlorobenzamide-5-aminobenzimidazole-2-yl) thioacetyl) mutilin* (**4a**). Synthesized according to the general procedure for 36 h. White solid; yield 94% (1.25 g). IR (KBr): 3422, 2933, 1726, 1655, 1603, 1544, 1445, 1406, 1353, 1272, 1153, 1117, 1016, 980, 749 cm^−1^; ^1^H-NMR (400 MHz, CDCl_3_) δ 10.98 (s, 1H), 8.20 (s, 2H), 7.67 (d, *J* = 6.0 Hz, 1H), 7.34 (dd, *J* = 38.3 Hz, 15.1, 4H), 6.41–6.25 (m, 1H), 5.70 (d, *J* = 7.8 Hz, 1H), 5.12 (dd, *J* = 70.7 Hz, 14.0, 2H), 4.10–4.01 (m, 1H), 3.85 (s, 2H), 3.26 (s, 1H), 2.24 (s, 1H), 2.20–2.05 (m, 2H), 2.00 (s, 1H), 1.97 (s, 1H), 1.93 (dd, *J* = 15.9 Hz, 8.4, 1H), 1.68 (d, *J* = 13.9 Hz, 1H), 1.57–1.48 (m, 2H), 1.45–1.16 (m, 9H), 1.11–0.95 (m, 4H), 0.80 (d, *J* = 2.7 Hz, 3H), 0.65 (d, *J* = 2.9 Hz, 3H); ^13^C-NMR (101 MHz, CDCl_3_) δ 216.00, 170.15, 167.49, 163.84, 148.04, 137.76, 134.32, 131.63, 130.63, 129.73, 129.38, 129.14, 126.29, 116.30, 73.61, 69.65, 59.39, 57.11, 44.43, 43.46, 42.98, 40.86, 35.72, 35.04, 34.78, 34.10, 33.44, 29.40, 25.86, 25.45, 23.83, 20.02, 15.81, 13.82, 13.18, 10.48; HRMS (ES) calcd [M + H]^+^ for C_36_H_42_N_3_O_5_SCl 664.2600, found 664.2598.

*14-O-((3-Chlorobenzamide-5-aminobenzimidazole-2-yl) thioacetyl) mutilin* (**4b**). Synthesized according to the general procedure for 40 h. White solid; yield 73% (0.97 g). IR (KBr): 3422, 2934, 1725, 1655, 1543, 1445, 1406, 1284, 1153, 1117, 1017, 980, 916 cm^−1^; ^1^H-NMR (400 MHz, CDCl_3_) δ 8.40 (s, 1H), 8.03 (s, 1H), 7.82 (s, 1H), 7.70 (d, *J* = 6.3 Hz, 1H), 7.40 (d, *J* = 7.0 Hz, 1H), 7.29 (s, 2H), 6.37–6.24 (m, 1H), 5.68 (d, *J* = 7.5 Hz, 1H), 5.08 (dd, *J* = 51.3 Hz, 14.0, 2H), 4.14–3.96 (m, 1H), 3.87 (q, *J* = 16.1 Hz, 2H), 3.26 (s, 1H), 2.20 (d, *J* = 17.1 Hz, 1H), 2.17–2.04 (m, 2H), 2.00 (s, 1H), 1.97 (s, 1H), 1.93 (dd, *J* = 15.4 Hz, 7.9, 1H), 1.67 (d, *J* = 14.2 Hz, 1H), 1.54 (d, *J* = 10.4 Hz, 1H), 1.50–1.11 (m, 9H), 1.03 (d, *J* = 20.1 Hz, 4H), 0.79 (s, 3H), 0.65 (s, 3H); ^13^C-NMR (101 MHz, CDCl_3_) δ 216.08, 170.17, 167.54, 163.89, 148.04, 137.76, 135.75, 133.86, 131.64, 130.76, 129.02, 126.50, 124.33, 116.28, 73.60, 69.76, 59.40, 57.11, 44.44, 43.49, 43.00, 40.87, 35.73, 35.03, 34.09, 33.46, 29.39, 29.10, 25.86, 25.47, 23.83, 20.03, 15.84, 13.82, 13.19, 10.48; HRMS (ES) calcd [M + H]^+^ for C_36_H_42_N_3_O_5_SCl 664.2600, found 664.2599.

*14-O-((4-Chlorobenzamide-5-aminobenzimidazole-2-yl) thioacetyl) mutilin* (**4c**). Synthesized according to the general procedure for 36 h. White solid; yield 89% (1.18 g). IR (KBr): 3373, 2934, 1727, 1654, 1598, 1544, 1486, 1445, 1285, 1153, 1116, 1099, 980, 938 cm^−1^; ^1^H-NMR (400 MHz, CDCl_3_) δ 7.97 (dd, *J* = 28.4 Hz, 21.8, 3H), 7.63 (dd, *J* = 52.2 Hz, 16.8 Hz, 2H), 7.40–7.21 (m, 2H), 6.11–5.97 (m, 1H), 5.51 (d, *J* = 6.8 Hz, 1H), 4.99 (dd, *J* = 74.7 Hz, 14.4, 2H), 4.45 (s, 1H), 4.14–4.02 (m, 2H), 3.37 (s, 1H), 2.35 (s, 1H), 2.20–2.10 (m, 1H), 2.04 (dd, *J* = 20.3 Hz, 9.6, 2H), 1.97 (s, 1H), 1.95–1.87 (m, 1H), 1.69–1.54 (m, 2H), 1.45 (s, 1H), 1.40–1.10 (m, 9H), 1.03–0.87 (m, 4H), 0.78 (s, 3H), 0.60 (s, 3H); ^13^C-NMR (101 MHz, CDCl_3_) δ 217.54, 167.31, 164.62, 149.95, 149.21, 141.07, 140.69, 136.66, 135.89, 134.34, 133.99, 132.77, 129.97, 128.84, 117.44, 115.78, 110.25, 73.06, 70.59, 60.19, 57.69, 45.40, 44.60, 41.99, 36.81, 34.45, 30.56, 29.15, 27.06, 24.91, 21.21, 16.52, 14.97, 11.95; HRMS (ES) calcd [M + H]^+^ for C_36_H_42_N_3_O_5_SCl 664.2600, found 664.2601.

*14-O-((2-Methylbenzamide-5-aminobenzimidazole-2-yl) thioacetyl) mutilin* (**4d**). Synthesized according to the general procedure for 40 h. White solid; yield 71% (0.91 g). IR (KBr): 3423, 2932, 1726, 1655, 1601, 1542, 1447, 1406, 1278, 1153, 1117, 1018, 981, 939, 917, 741 cm^−1^; ^1^H-NMR (400 MHz, CDCl_3_) δ 8.32 (s, 1H), 7.96–7.72 (m, 1H), 7.35 (dd, *J* = 57.9 Hz, 6.5, 3H), 7.18 (s, 1H), 6.80 (d, *J* = 70.7 Hz, 1H), 6.35 (ddd, *J* = 15.6 Hz, 10.2, 8.3, 1H), 5.69 (d, *J* = 7.0 Hz, 1H), 5.12 (dd, *J* = 49.2 Hz, 14.2, 2H), 3.82 (s, 2H), 3.26 (s, 1H), 2.44 (s, 3H), 2.17 (dd, *J* = 43.0 Hz, 5.2, 3H), 1.99 (s, 1H), 1.89 (dd, *J* = 22.6 Hz, 14.6, 1H), 1.66 (s, 1H), 1.55 (d, *J* = 9.6 Hz, 1H), 1.31 (ddt, *J* = 19.6 Hz, 14.3, 9.5, 10H), 1.01 (d, *J* = 10.8 Hz, 4H), 0.80 (d, *J* = 6.8 Hz, 3H), 0.64 (d, *J* = 6.8 Hz, 3H); ^13^C-NMR (101 MHz, CDCl_3_) δ 216.06, 167.50, 147.94, 137.77, 135.53, 135.22, 132.02, 130.26, 129.29, 127.83, 125.71, 124.95, 116.31, 73.61, 69.61, 64.57, 59.40, 57.11, 44.43, 43.44, 42.97, 40.85, 35.72, 35.03, 34.02, 33.44, 29.56, 29.39, 25.85, 25.44, 23.83, 20.03, 18.84, 15.80, 13.82, 13.18, 10.48; HRMS (ES) calcd [M + H]^+^ for C_37_H_45_N_3_O_5_S 644.3162, found 644.3160.

*14-O-((3-Methylbenzamide-5-aminobenzimidazole-2-yl) thioacetyl) mutilin* (**4e**). Synthesized according to the general procedure for 36 h. White solid; yield 76% (0.98 g). IR (KBr): 3395, 2932, 1726, 1649, 1602, 1541, 1446, 1406, 1284, 1153, 1117, 1018, 981, 938, 917, 740 cm^−1^; ^1^H-NMR (400 MHz, CDCl_3_) δ 8.28 (d, *J* = 5.4 Hz, 1H), 8.12 (s, 1H), 7.63 (dd, *J* = 10.1 Hz, 6.6, 2H), 7.40–7.26 (m, 2H), 7.07 (s, 1H), 6.32 (dd, *J* = 17.4 Hz, 11.1, 1H), 5.67 (d, *J* = 8.4 Hz, 1H), 5.29–4.93 (m, 2H), 3.88 (s, 2H), 3.24 (d, *J* = 6.1 Hz, 1H), 2.32 (s, 3H), 2.20 (d, *J* = 6.8 Hz, 1H), 2.11 (d, *J* = 7.8 Hz, 2H), 1.97 (d, *J* = 3.0 Hz, 1H), 1.90 (dd, *J* = 16.0 Hz, 8.4, 1H), 1.64 (s, 1H), 1.53 (d, *J* = 10.2 Hz, 2H), 1.50–1.10 (m, 9H), 1.00 (s, 4H), 0.78 (d, *J* = 6.8, 3H), 0.63 (d, *J* = 6.8, 3H); ^13^C-NMR (101 MHz, CDCl_3_) δ216.08, 167.31, 165.55, 147.94, 137.79, 137.70, 133.94, 132.00, 131.62, 129.90, 127.67, 126.87, 123.15, 116.27, 114.94, 73.60, 69.61, 64.57, 57.11, 44.43, 43.44, 42.97, 40.85, 35.71, 35.03, 34.15, 33.44, 29.57, 29.39, 25.85, 25.46, 23.82, 20.39, 18.17, 15.78, 13.80, 10.47; HRMS (ES) calcd [M + H]^+^ for C_37_H_45_N_3_O_5_S 644.3162 ,found 644.3154.

*14-O-((4-Methylbenzamide-5-aminobenzimidazole-2-yl) thioacetyl) mutilin* (**4f**). Synthesized according to the general procedure for 40 h. White solid; yield 61% (0.79 g). IR (KBr): 3422, 2928, 1726, 1635, 1609, 1541, 1446, 1425, 1297, 1180, 1117, 1019, 980, 940, 912, 747 cm^−1^; ^1^H-NMR (400 MHz, CDCl_3_) δ 8.43 (d, *J* = 43.5 Hz, 1H), 8.34 (d, *J* = 7.7 Hz, 1H), 7.73–7.42 (m, 3H), 7.17 (t, *J* = 7.5 Hz, 1H), 7.05 (d, *J* = 8.3 Hz, 1H), 6.40 (dd, *J* = 17.4 Hz, 11.0, 1H), 5.75 (d, *J* = 8.3 Hz, 1H), 5.18 (dd, *J* = 51.6 Hz, 14.2, 2H), 4.09 (d, *J* = 10.7 Hz, 3H), 3.94 (d, *J* = 4.8 Hz, 2H), 3.31 (s, 1H), 2.41–2.23 (m, 2H), 2.23–2.12 (m, 2H), 2.05 (s, 1H), 2.04 (s, 1H), 1.96 (dd, *J* = 15.8 Hz, 8.4, 1H), 1.73 (t, *J* = 13.6 Hz, 1H), 1.61 (d, *J* = 9.9 Hz, 2H), 1.55–1.20 (m, 9H), 1.06 (s, 4H), 0.86 (d, *J* = 6.9 Hz, 3H), 0.70 (d, *J* = 6.8 Hz, 3H); ^13^C-NMR (101 MHz, CDCl_3_) δ 215.94,167.41, 167.32, 162.45, 156.29, 147.67, 137.80, 132.32, 131.53, 120.79, 116.26, 110.62, 73.61, 69.51, 59.39, 57.11, 55.30, 44.43, 43.44, 42.97, 40.85, 35.73, 35.04, 34.17, 33.44, 29.41, 25.86, 25.43, 23.83, 20.02,18.17, 15.76, 13.79, 13.18, 10.46; HRMS (ES) calcd [M + H]^+^ for C_37_H_45_N_3_O_5_S 644.3162, found 644.3183.

*14-O-((2-Methoxybenzamide-5-aminobenzimidazole-2-yl) thioacetyl) mutilin* (**4g**). Synthesized according to the general procedure for 36 h. White solid; yield 68% (0.90 g). IR (KBr): 3357, 2934, 1729, 1653, 1600, 1551, 1457, 1406, 1292, 1237, 1162, 1117, 1019, 980, 938, 916, 756 cm^−1^; ^1^H-NMR (400 MHz, CDCl_3_) δ 8.04 (s, 1H), 7.88 (d, *J* = 6.7 Hz, 2H), 7.57–7.20 (m, 4H), 6.11–5.98 (m, 1H), 5.53 (d, *J* = 7.1 Hz, 1H), 5.01 (dd, *J* = 74.0 Hz, 14.3, 2H), 4.47 (s, 1H), 4.09 (s, 2H), 3.38 (s, 1H), 2.39 (s, 3H), 2.36 (s, 1H), 2.16 (d, *J* = 11.1 Hz, 1H), 2.11–1.87 (m, 4H), 1.62 (dd, *J* = 25.4 Hz, 12.7, 2H), 1.47 (s, 1H), 1.42–1.11 (m, 9H), 0.99 (d, *J* = 28.2 Hz, 4H), 0.80 (d, *J* = 5.3 Hz, 3H), 0.62 (d, *J* = 3.2 Hz, 3H); ^13^C-NMR (101 MHz, CDCl_3_) δ 217.68, 167.48, 165.71, 149.15, 141.90, 141.22, 140.67, 136.04, 134.44, 132.93, 129.44, 128.21, 117.52, 115.93, 115.52, 102.88, 73.21, 70.74, 60.34, 57.84, 45.55, 44.75, 43.71, 42.14, 36.96, 34.59, 30.71, 29.28, 27.21, 25.06, 21.60, 16.68, 15.12, 14.69, 12.10; HRMS (ES) calcd [M + H]^+^ for C_37_H_45_N_3_O_6_S 660.3145, found 660.3149.

*14-O-((3-Methoxybenzamide-5-aminobenzimidazole-2-yl) thioacetyl) mutilin* (**4h**). Synthesized according to the general procedure for 40 h. White solid; yield 57% (0.75 g). IR (KBr): 3385, 2936, 1727, 1654, 1598, 1541, 1447, 1349, 1287, 1154, 1117, 1039, 980, 937, 918, 804 cm^−1^; ^1^H-NMR (400 MHz, CDCl_3_) δ 8.30 (s, 1H), 8.10 (s, 1H), 7.38 (s, 2H), 7.36 (s, 1H), 7.27 (t, *J* = 7.8, 1H), 6.98 (d, *J* = 6.0, 1H), 6.32 (dd, *J* = 17.4 Hz, 11.1, 1H), 5.68 (d, *J* = 8.3 Hz, 1H), 5.10 (dd, *J* = 43.3 Hz, 14.2, 2H), 4.05 (dt, *J* = 7.1 Hz, 6.2, 1H), 3.84 (d, *J* = 15.7 Hz, 2H), 3.76 (s, 3H), 3.24 (s, 1H), 2.29–2.17 (m, 1H), 2.16–2.04 (m, 2H), 1.97 (d, *J* = 1.0 Hz, 1H), 1.94–1.85 (m, 1H), 1.66 (d, *J* = 14.4 Hz, 1H), 1.53 (d, *J* = 9.8 Hz, 1H), 1.44–1.17 (m, 8H), 1.00 (s, 4H), 0.79 (d, *J* = 6.7 Hz, 3H), 0.64 (d, *J* = 6.7 Hz, 4H); ^13^C-NMR (101 MHz, CDCl_3_) δ 216.18, 170.22, 167.42, 165.11, 158.89, 147.98, 137.72, 135.42, 131.85, 128.78, 117.97, 116.89, 116.30, 111.55, 76.31,73.56, 69.58, 59.42, 57.08, 54.47, 44.41, 43.37, 42.94, 40.82, 35.69, 34.99, 34.07, 33.44, 29.36, 25.82, 25.42, 23.80, 20.06, 15.80, 13.80, 13.19, 10.51; HRMS (ES) calcd [M + H]^+^ for C_37_H_45_N_3_O_6_S 660.3145, found 660.3098.

*14-O-((4-Methoxybenzamide-5-aminobenzimidazole-2-yl) thioacetyl) mutilin* (**4i**). Synthesized according to the general procedure for 36 h. White solid; yield 58% (0.76 g). IR (KBr): 3386, 2932, 1725, 1647, 1606, 1544, 1508, 1458, 1406, 1351, 1286, 1254, 1179, 1119, 1076, 980, 939, 917, 762 cm^−1^; ^1^H-NMR (400 MHz, CDCl_3_) δ 8.21 (s, 1H), 8.06 (d, *J* = 24.1 Hz, 1H), 7.81 (d, *J* = 8.8 Hz, 2H), 7.24 (s, 1H), 6.86 (d, *J* = 8.7 Hz, 2H), 6.32 (dd, *J* = 17.4 Hz, 11.0, 1H), 5.67 (d, *J* = 8.4 Hz, 1H), 5.09 (dd, *J* = 43.1 Hz, 14.2, 2H), 3.87 (d, *J* = 3.7 Hz, 2H), 3.78 (s, 3H), 3.25 (s, 1H), 2.20 (dd, *J* = 13.7 Hz, 7.1, 1H), 2.10 (dt, *J* = 17.5 Hz, 7.3, 2H), 1.97 (s, 1H), 1.97 (s, 1H), 1.90 (dd, *J* = 16.0 Hz, 8.5, 1H), 1.66 (d, *J* = 14.0 Hz, 1H), 1.54 (d, *J* = 7.1 Hz, 1H), 1.49–1.12 (m, 9H), 0.99 (s, 4H), 0.78 (d, *J* = 6.9 Hz, 3H), 0.64 (d, *J* = 6.9 Hz, 3H);^13^C-NMR (101 MHz, CDCl_3_) δ 216.05, 167.41, 164.80, 161.49, 147.85, 137.78, 132.08, 128.05, 126.11, 116.27, 112.99,76.23, 73.60, 69.61, 59.39, 57.11, 54.45, 44.43, 43.45, 42.98, 40.95,40.85, 35.72, 35.03, 34.15, 33.45, 29.39, 25.85, 25.45, 23.82, 20.02, 15.79, 13.80, 13.18, 10.47; HRMS (ES) calcd [M + H]^+^ for C_37_H_45_N_3_O_6_S 660.3145, found 660.3143.

*14-O-((Cyclohexanecarboxamide-5-aminobenzimidazole-2-yl) thioacetyl) mutilin* (**4j**). Synthesized according to the general procedure for 36 h. White solid; yield 81% (1.03 g). IR (KBr): 3365, 2931, 1728, 1664, 1601, 1541, 1448, 1407, 1345, 1277, 1208, 1152, 1117, 1018, 980, 938, 917, 807 cm^−1^; ^1^H-NMR (400 MHz, CDCl_3_) δ 8.16 (s, 1H), 7.61 (s, 1H), 7.39 (d, *J* = 7.9 Hz, 1H), 6.93 (s, 1H), 6.39 (dd, *J* = 17.4 Hz, 11.0, 1H), 5.74 (d, *J* = 8.4 Hz, 1H), 5.33–5.00 (m, 3H), 3.96 (d, *J* = 6.1 Hz, 1H), 3.30 (d, *J* = 4.6 Hz, 1H), 2.39–2.10 (m, 4H), 2.08–1.95 (m, 4H), 1.69 (dd, *J* = 72.2 Hz, 26.0, 10H), 1.41–1.15 (m, 9H), 1.05 (s, 4H), 0.86 (d, *J* = 6.9 Hz, 3H), 0.71 (d, *J* = 6.9 Hz, 3H); ^13^C-NMR (101 MHz, CDCl_3_) δ 216.03, 173.89, 167.29, 147.69, 137.80, 131.96, 129.91, 127.84, 116.25, 73.61, 69.53, 59.40, 57.12, 52.41, 45.65, 44.44, 43.44, 42.97, 40.86, 35.73, 35.04, 34.17, 33.46, 29.41, 28.82, 25.86, 25.44, 24.70, 23.83, 20.03, 18.18, 15.76, 13.80, 10.46; HRMS (ES) calcd [M + H]^+^ for C_36_H_49_N_3_O_5_S 636.3495, found 636.3497.

*14-O-(((1H-Pyrrole-2-carboxamide)-5-aminobenzimidazole-2-yl) thioacetyl) mutilin* (**4k**). Synthesized according to the general procedure for 48 h. White solid; yield 76% (0.94 g). IR (KBr): 3374, 2933, 1724, 1638, 1601, 1554, 1444, 1408, 1352, 1271, 1191, 1151, 1117, 1017, 980, 937, 917, 746 cm^−1^; ^1^H-NMR (400 MHz, CDCl_3_) δ 9.95 (s, 1H), 8.09 (s, 1H), 7.85 (s, 1H), 7.30 (s, 1H), 7.09–6.85 (m, 2H), 6.75 (s, 1H), 6.29 (dd, *J* = 17.3 Hz, 11.1, 1H), 6.19 (s, 1H), 5.67 (d, *J* = 8.0 Hz, 1H), 5.05 (dd, *J* = 41.0 Hz, 14.2, 2H), 3.87 (s, 2H), 3.25 (s, 1H), 2.19 (d, *J* = 6.7 Hz, 1H), 2.16–2.02 (m, 2H), 1.98 (d, *J* = 2.7 Hz, 1H), 1.89 (dd, *J* = 15.7 Hz, 7.9, 1H), 1.65 (d, *J* = 13.8 Hz, 1H), 1.54 (s, 1H), 1.53–1.09 (m, 9H), 1.06–0.92 (m, 4H), 0.77 (d, *J* = 6.7 Hz, 3H), 0.62 (d, *J* = 6.7 Hz, 3H); ^13^C-NMR (101 MHz, CDCl_3_) δ 216.21, 167.56,158.57, 158.51, 147.60, 147.23, 137.70, 131.83, 124.91, 121.63, 116.29,109.28, 109.15,76.21, 73.59, 69.69, 57.10, 52.41,49.03, 44.43, 43.48, 42.95, 40.84, 35.71, 35.00, 34.47, 33.47, 29.36, 25.83, 25.40, 23.82, 15.76, 13.80, 10.49; HRMS (ES) calcd [M + H]^+^ for C_34_H_43_N_3_O_5_S 619.2951, found 619.2953.

*14-O-(((1H-Indole-2-carboxamide)-5-aminobenzimidazole-2-yl) thioacetyl) mutilin* (**4l**). Synthesized according to the general procedure for 48 h. White solid; yield 81% (1.08 g). IR (KBr): 3380, 2934, 1725, 1648, 1601, 1545, 1445, 1418, 1342, 1308, 1276, 1240, 1149, 1117, 1017, 980, 937, 917, 812, 747, 628 cm^−1^; ^1^H-NMR (400 MHz, CDCl_3_) δ 12.55 (s, 1H), 11.73 (d, *J* = 11.3 Hz, 1H), 10.16 (d, *J* = 22.0 Hz, 1H), 8.05 (s, 1H), 7.68 (d, *J* = 8.0 Hz, 1H), 7.53–7.46 (m, 1H), 7.43 (d, *J* = 1.7 Hz, 2H), 7.22 (t, *J* = 7.6 Hz, 1H), 7.07 (t, *J* = 7.5 Hz, 1H), 6.07 (dd, *J* = 17.1 Hz, 11.9, 1H), 5.53 (d, *J* = 8.1 Hz, 1H), 5.02 (dd, *J* = 48.6 Hz, 14.5, 2H), 4.48 (t, *J* = 5.9 Hz, 1H), 4.12 (d, *J* = 10.1 Hz, 1H), 3.38 (s, 1H), 2.37 (s, 1H), 2.17 (dd, *J* = 18.6 Hz, 10.9, 1H), 2.04 (d, *J* = 9.4 Hz, 1H), 1.99 (s, 1H), 1.93 (dd, *J* = 16.0 Hz, 8.3, 1H), 1.62 (s, 2H), 1.46 (d, *J* = 7.4 Hz, 1H), 1.43–1.11 (m, 9H), 0.97 (d, *J* = 6.2 Hz, 4H), 0.80 (d, *J* = 6.8 Hz, 3H), 0.63 (t, *J* = 6.3 Hz, 3H); HRMS (ES) calcd [M + H]^+^ for C_38_H_45_N_4_O_5_S 669.3107, found 669.3109.

### 3.2. Biological Evaluation

#### 3.2.1. MIC Testing

The MIC for the synthesized compounds (**3** and **4a**–**l**) and tiamulin fumarate used as a reference drug were determined using the agar dilution method according to the National Committee for Clinical Laboratory Standards (NCCLS). Tested compounds were dissolved in 25%–40% DMSO to a solution with a concentration of 1280 µg/mL, whereas tiamulin fumarate was directly dissolved in 10 mL of distilled water at the same concentration as the tested compounds. All the solutions were then diluted twofold with sterile water to provide 11 dilutions (final concentration is 0.625 µg/mL). A 2 mL volume of the 2-fold serial dilution of each test compound/drug was incorporated into 18 mL of hot Muellere-Hinton agar medium. Inoculums, including five Gram (+), *S. aureus*, MRSA, *S. vitulinus*, *S. warneri*, *S. haemolyticus*, and one Gram (−), *E. coli* were prepared from blood slants and adjusted to approximately 10^5^–10^6^ CFU/mL with sterile saline (0.90% NaCl). A 10 µL amount of bacterial suspension was spotted onto Muellere-Hinton agar plates containing serial dilutions of the compounds/drug. The plates were incubated at 36.5 °C for 24–48 h. The same procedure was repeated in triplicate.

#### 3.2.2. Oxford Cup Assays

The procedure for Oxford cup assays was the same as that previously reported [13]. Briefly, inoculums were prepared in 0.9% saline using McFarland standard and spread uniformly on nutrient agar plates. The 320 and 160 µg/mL of all the tested compounds were added individually into the Oxford cups that were placed at equal distances above the agar surfaces. The zone of inhibition for each concentration was measured after a 24–36 h incubation at 37 °C. The same procedure was repeated in triplicate.

#### 3.2.3. Inhibition of the Bacterial Growth

The inhibition activities of test compounds were evaluated by measuring the absorbance of the bacterial suspension. In brief, inoculums were prepared from blood slants and adjusted to approximately 10^6^ CFU/mL in Muellere-Hinton broth. The 0.0625, 0.125, 0.25, 0.5, 1, and 2 µM of tested compounds were prepared and added to the prepared inoculums. A sample of inoculum with no compound was used as control. The absorbance of bacterial suspension at 450 nm was measured using an UV spectrophotometer after 3.5 h of incubation, and the inhibition was calculated as 1 − (A^P^/A^0^), where A^p^ and A^0^ are the absorbance of bacterial suspension in the presence and absence of compounds, respectively. The same procedure was independently repeated in triplicate.

#### 3.2.4. Cytochrome P450 Inhibition Assay

Compound **3** was screened for its ability to inhibit cytochrome P450 using a cocktail assay described previously [19,20]. Briefly, 50 μL of compound **3** was added to 96-well plates, with final concentrations of 0.05, 0.1, 0.5, 1, 10, 50, and 100 μM, respectively. Twenty microliters of human liver microsomes (final concentration of 0.3 mg/mL) and 20 μL of probe substrates (Table 3) in 0.1 M Tris were added. After preincubation at 37 °C for 10 min, 10 μL of NADPH was added (final concentration of 1 mM), and the reaction started to incubate at the same temperature for 15 min. The reactions were then quenched by the addition of 100 μL of acetonitrile with a mixture of propranolol and nadolol (50 nM) used as an internal standard. After reactions were finished, the plates were centrifuged and the supernatants were analyzed for the five metabolites (Table 3) by LC/MS/MS.

## 4. Conclusions

Novel antibacterial pleuromutilin derivatives were synthesized by introduction of the substituted benzimidazole moieties to its C22 side. These derivatives were initially evaluated for their in vitro antibacterial activities against *E. coli*, *S. aureus*, MRSA, *S. vitulinus*, *S. warneri*, and *S. haemolyticus*. Compounds **3** and **4j**–**l** exhibited promising in vitro antibacterial effects against all the pathogens except *E. coli*. The further evaluation of compound **3** displayed higher growth inhibition with GI_50_ values of 0.0987 and 0.1748 μM against *S. aureus* and MRSA, respectively. The CYP450 inhibition assay of compound **3** showed intermediate in vitro inhibitory potency for CYP3A4, a member of the CYP450 family with the most common and the most versatile function. This study indicates that further designing new pleuromutilin derivatives should be considered to improve the CYP450 inhibition profile.

## Figures and Tables

**Table 1:** In vitro antibacterial activity (MIC) of the synthesized pleuromutilin derivatives.

Compound No.	MIC (µg/mL)					
	*E. coli*	*S. aureus*	MRSA	*S. vitulinus*	*S. warneri*	*S. haemolyticus*
**3**	10	0.156	0.156	0.156	0.156	0.156
**4a**	40	40	40	40	40	5
**4b**	80	40	20	10	20	20
**4c**	40	40	40	40	40	40
**4d**	80	10	40	40	40	40
**4e**	80	40	40	40	40	40
**4f**	80	40	40	40	20	40
**4g**	80	40	40	40	40	10
**4h**	40	40	40	40	40	10
**4i**	40	40	20	40	20	5
**4j**	20	5	1.25	1.25	2.5	10
**4k**	20	1.25	1.25	1.25	5	1.25
**4l**	20	10	10	5	2.5	10
Tiamulin	1.25	0.156	0.156	0.156	0.156	0.156

**Table 2:** Zone of inhibition for *E. coli*, *S. aureus*, MRSA, *S. vitulinus*, *S. warneri*, and *S. haemolyticus* in mm.

Compound	*E. coli*		*S. aureus*		MRSA		*S. vitulinus*		*S. warneri*		*S. haemolyticus*	
	320	160	320	160	320	160	320	160	320	160	320	160
**3**	25.52	21.38	29.56	27.28	30.56	25.42	29.56	25.48	32.28	28.02	33.04	30.28
**4a**	11.28	0.00	12.44	9.02	20.16	15.88	19.22	13.78	22.38	21.58	24.92	20.38
**4b**	0.00	10.38	15.84	12.28	21.68	17.26	23.82	20.48	19.48	16.46	20.38	20.30
**4c**	17.66	17.16	16.46	14.30	15.46	14.58	21.76	20.54	22.24	19.18	20.95	16.95
**4d**	0.00	0.00	17.84	14.72	19.70	16.30	19.12	18.18	22.28	20.60	18.36	17.38
**4e**	0.00	0.00	19.36	17.88	27.76	25.58	20.26	18.10	21.46	20.82	20.80	18.32
**4f**	8.64	0.00	17.84	16.72	17.32	14.06	15.08	14.00	22.94	22.86	20.72	16.20
**4g**	9.38	0.00	18.34	13.74	21.54	19.12	18.00	16.24	20.10	18.54	25.58	24.30
**4h**	18.02	15.32	18.72	15.88	19.70	16.30	21.66	19.78	19.78	17.92	28.41	25.41
**4i**	17.72	14.22	21.48	18.62	25.70	21.32	18.20	17.62	21.78	18.58	32.86	23.04
**4j**	19.78	17.44	27.25	15.42	31.90	28.00	29.10	25.98	27.69	24.72	26.36	23.00
**4k**	20.06	18.54	28.86	26.62	30.68	26.54	27.00	24.68	25.54	21.22	29.88	25.52
**4l**	20.48	17.36	24.72	20.88	21.58	19.76	26.54	22.06	29.56	25.36	25.12	20.80
Tiamulin	27.10	25.84	28.16	25.12	30.48	28.14	30.94	18.58	30.52	22.42	31.48	28.80

**Table 3:** Some experiment parameters and cytochrome P450 inhibition.

P450 Isozyme	Probe	Final Concentrations (mM) ^a^	Metabolite	IC_50_ (μM)
CYP1A2	Phenacetin	45	Acetaminophen	>50
CYP2C19	Mephenytoin	55	4-OH-*S*-mephenytoin	>50
CYP2D6	Dextromethorphan	10	Dextrophan	>100
CYP2C9	Diclofenac	10	4-OH-diclofenac	10.70
CYP3A4	Midazolam	5	1-OH-midazolam	1.69

^a^ The final concentrations of probe were set according to [17].

## Supplementary Materials

Supplementary materials can be accessed at: http://www.mdpi.com/1420-3049/21/11/1488/s1.
