# Supplementary material for: Synthesis and Pharmacological Evaluation of Novel Pleuromutilin Derivatives with Substituted Benzimidazole Moieties
Source: Molecules. 2016 Nov 8;21(11):1488. doi: 10.3390/molecules21111488 (PMC6273978; doi:10.3390/molecules21111488)

Supplementary Materials: Synthesis and Pharmacological Evaluation of Novel Pleuromutilin Derivatives with Substituted Benzimidazole Moieties

Xin Ai , Xiuying Pu, Yunpeng Yi, Yu Liu, Shuijin Xu, Jianping Liang and Ruofeng Shang

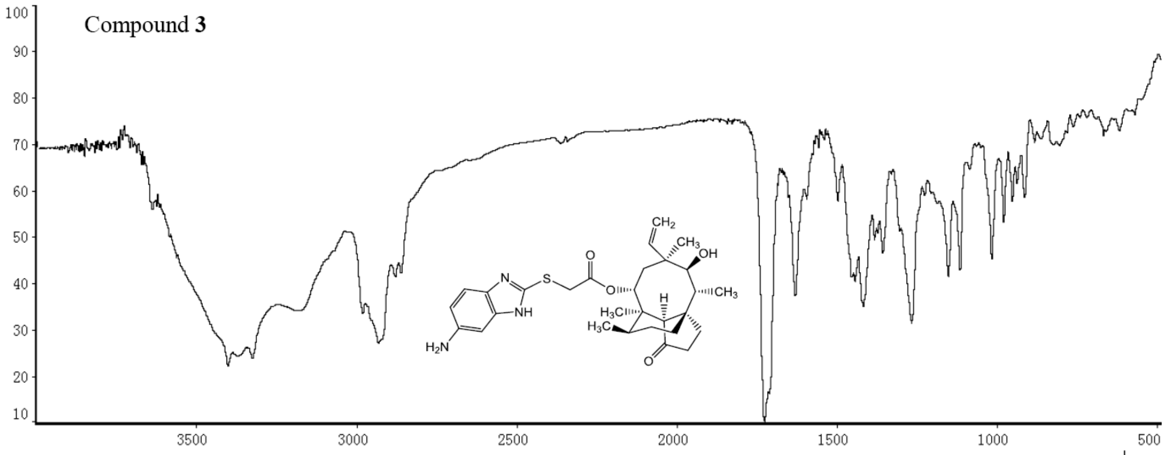

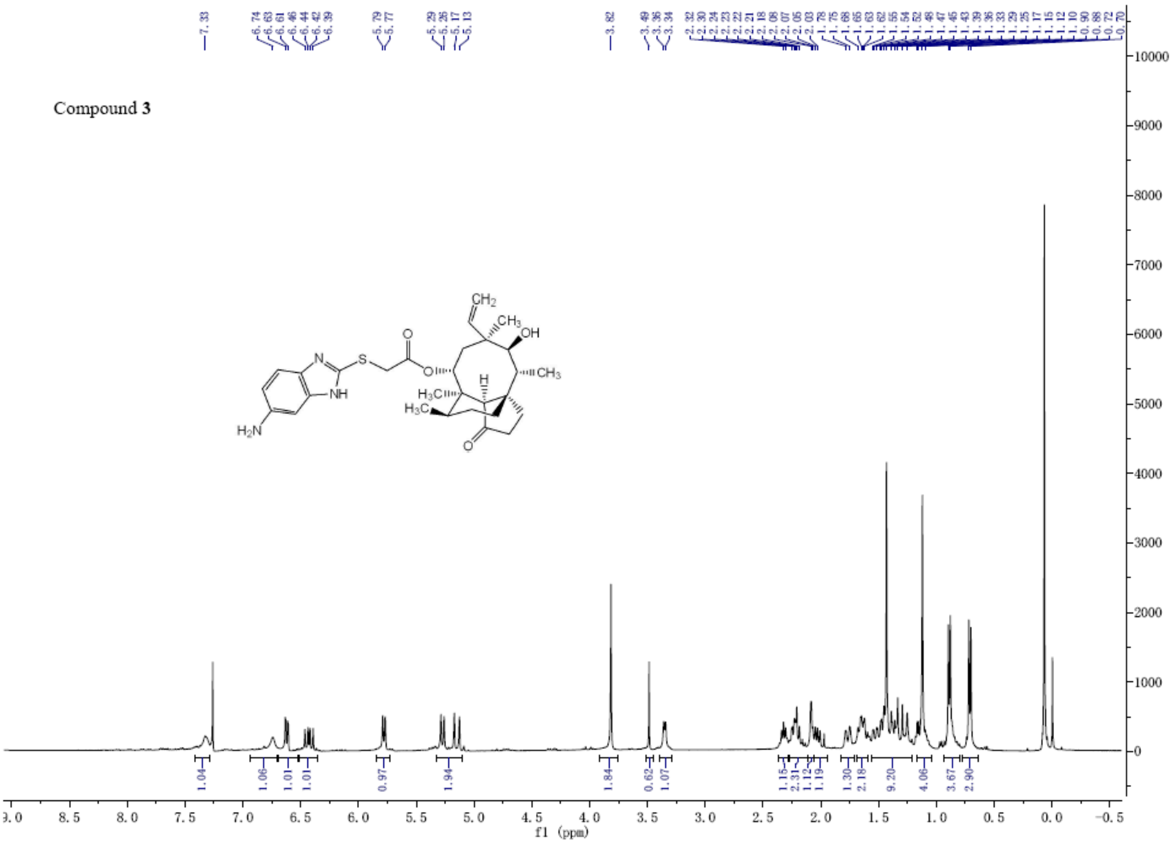

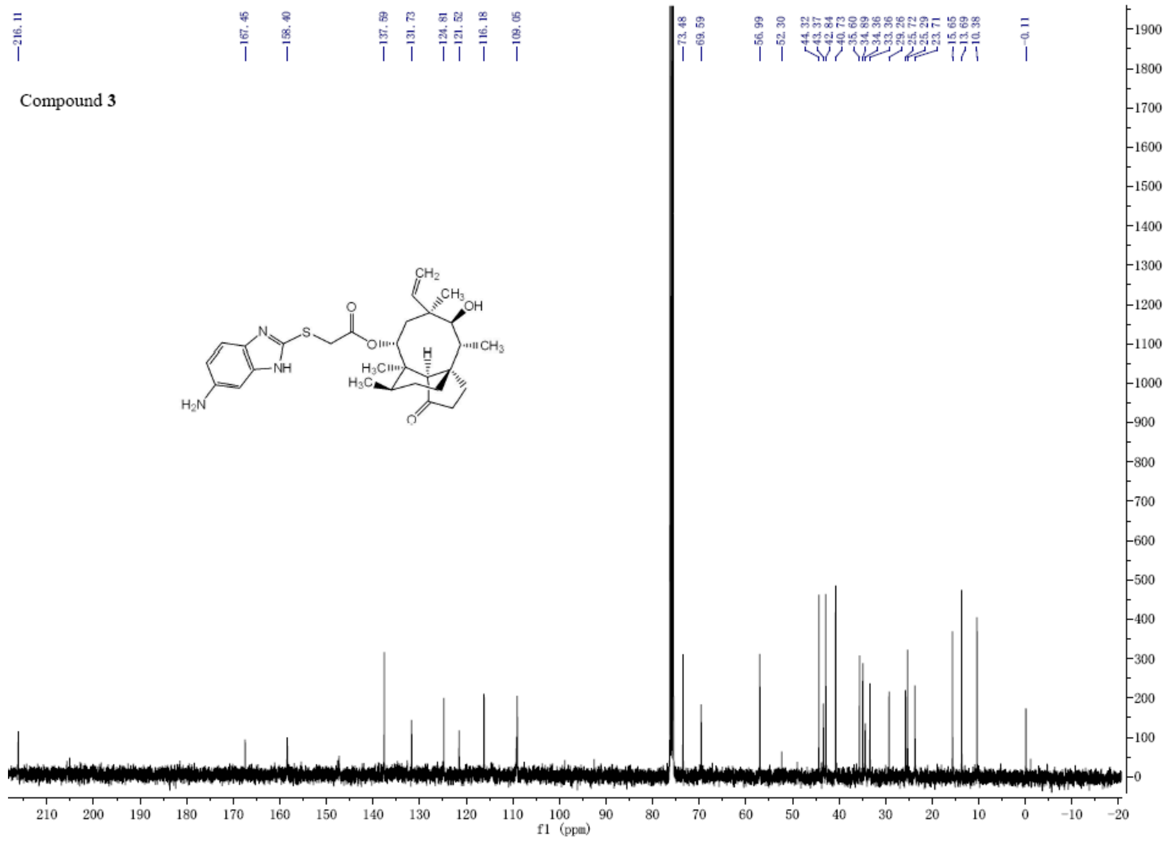
Compound **3**
f1 (ppm)

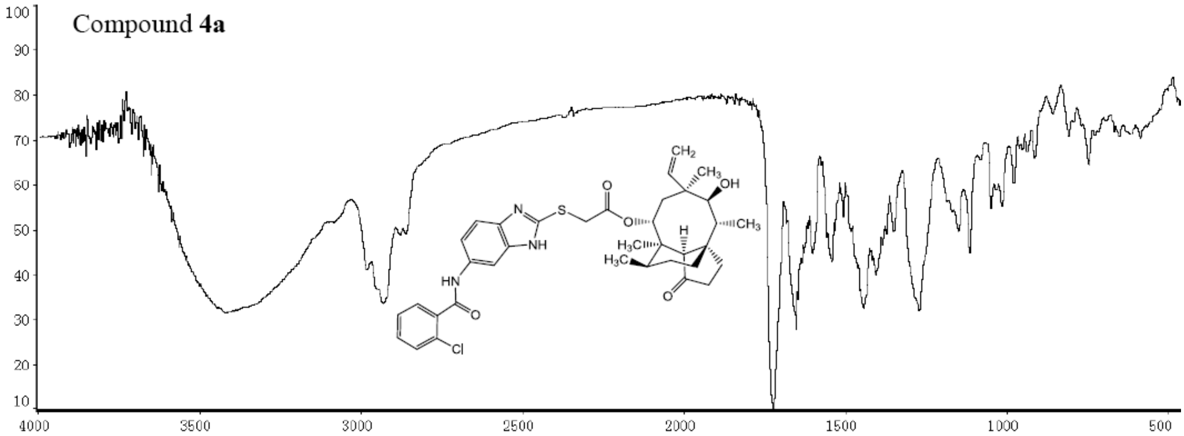
Compound **4a**

Compound **4a**

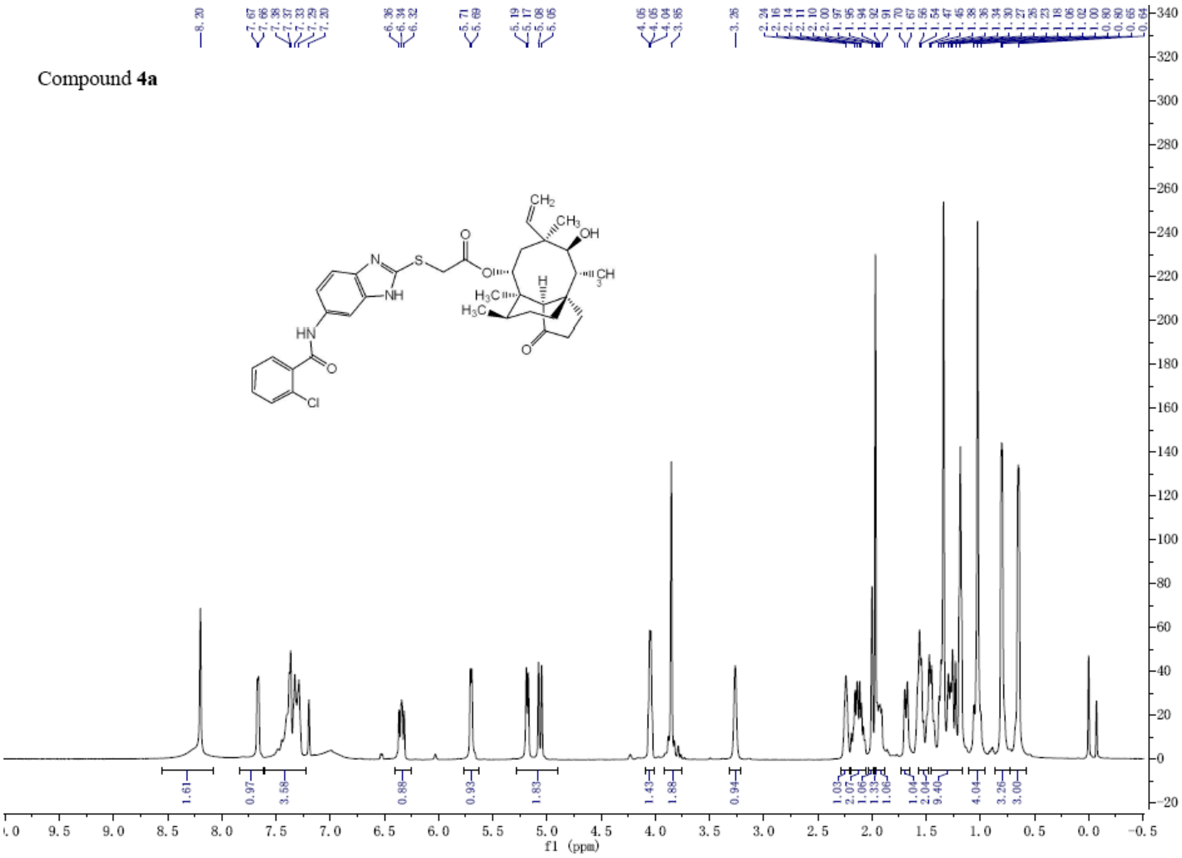

Compound **4a**

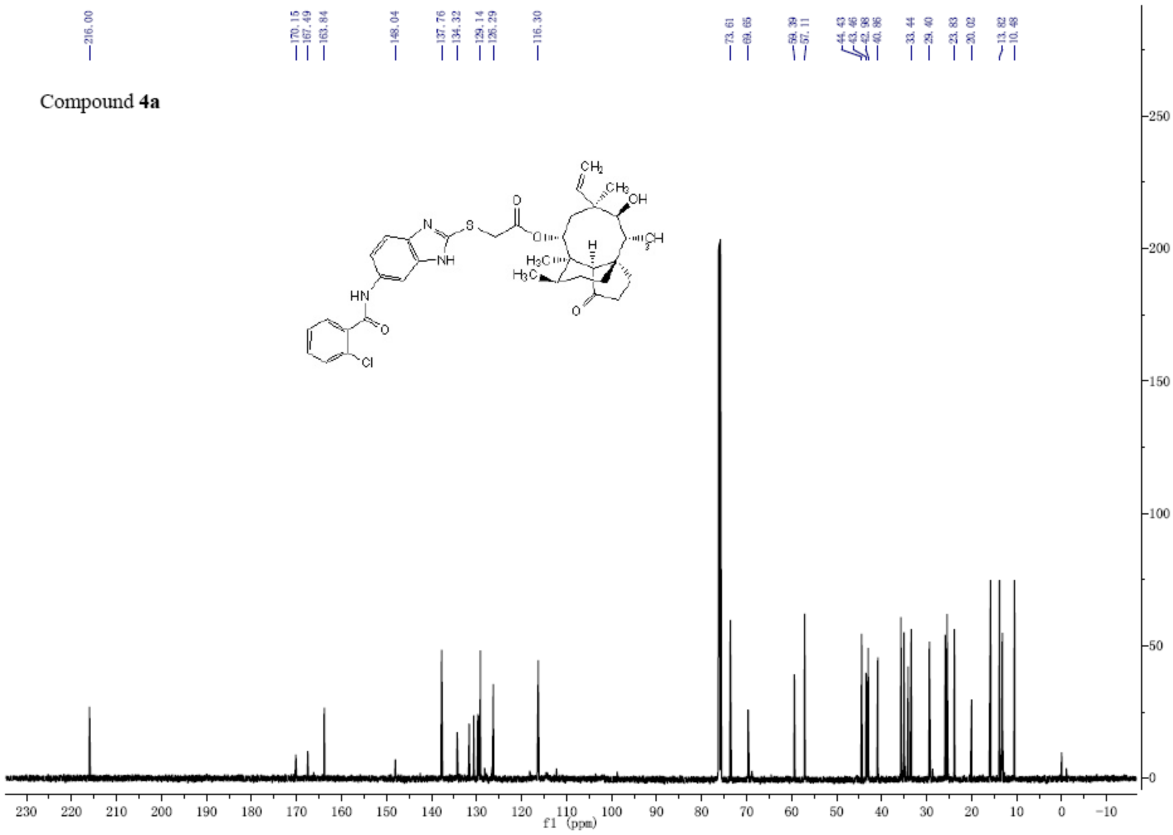

Compound **4b**

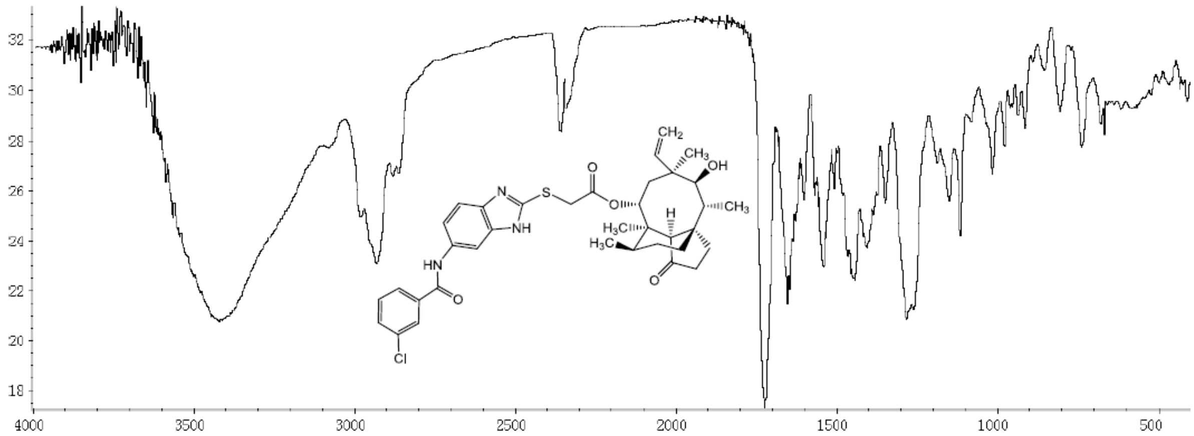

Compound **4b**

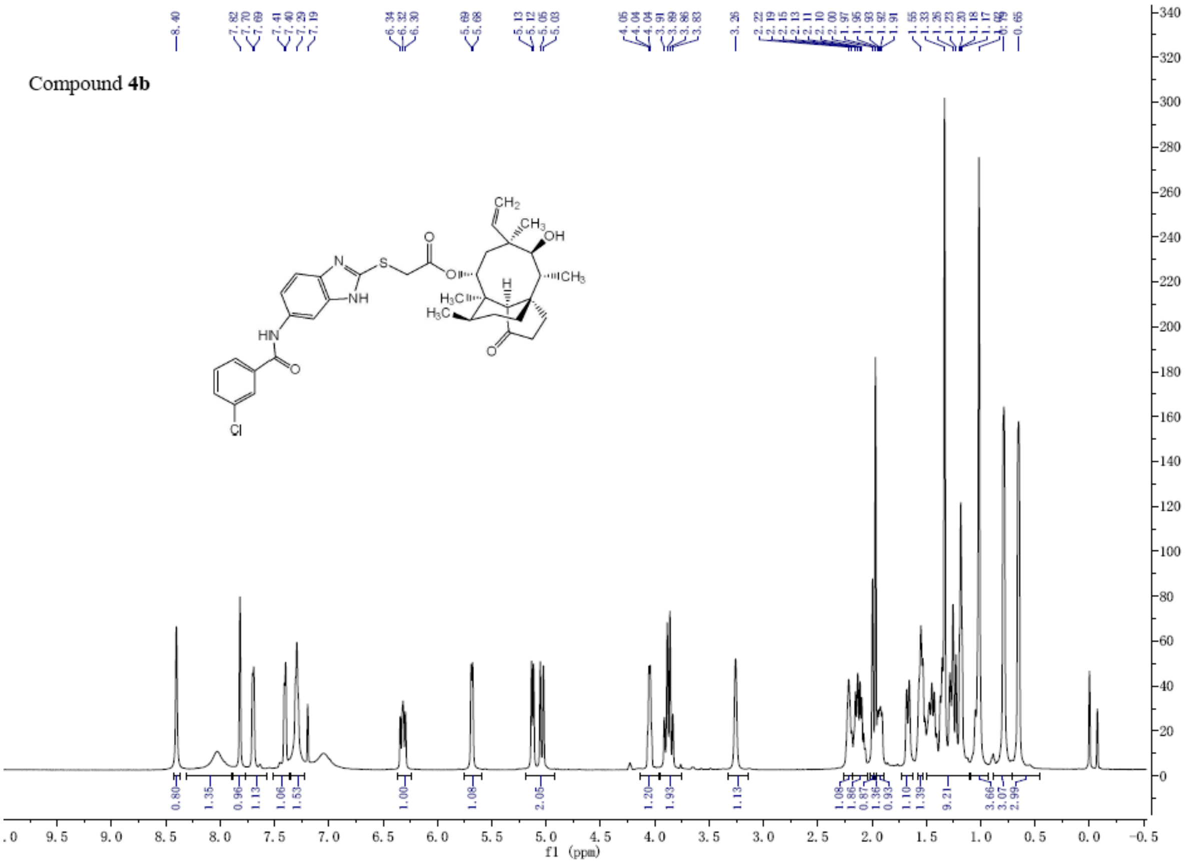

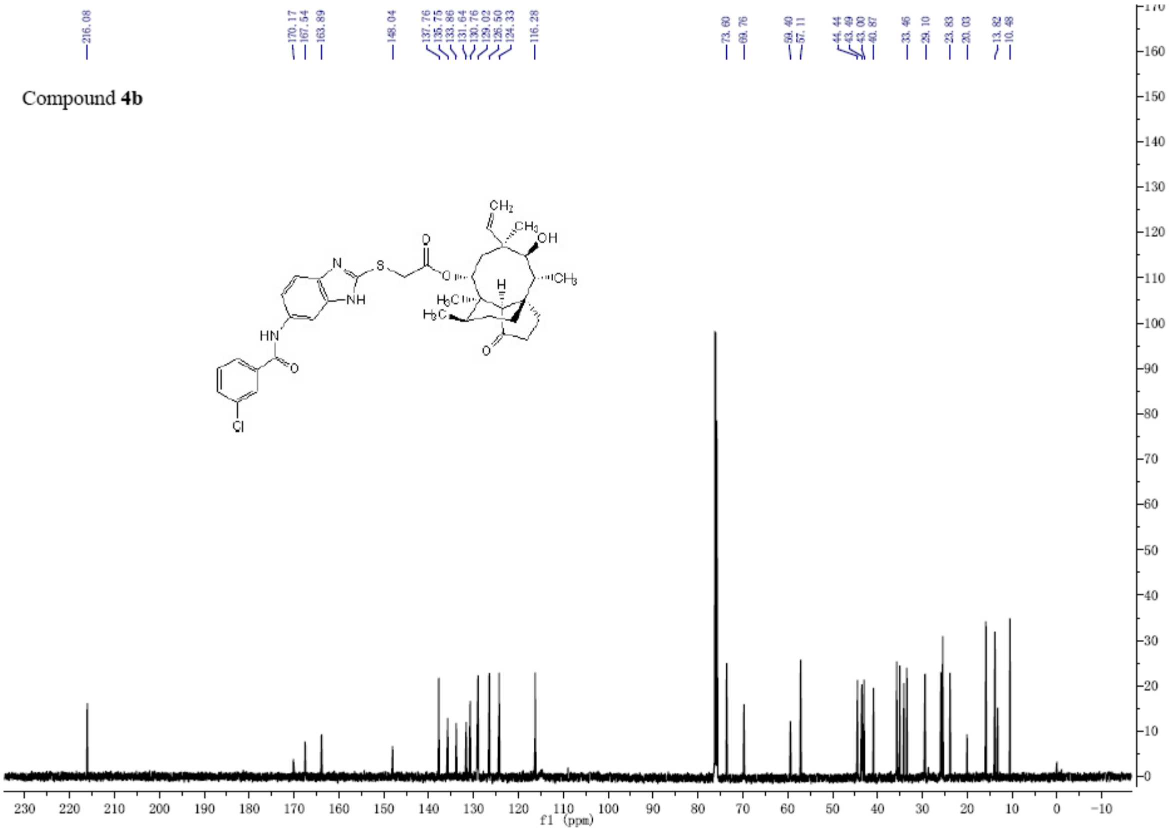
Compound **4b**
216.08
170.17
167.54
163.89
148.04
137.76
135.75
133.86
131.64
130.76
129.02
126.50
124.33
116.28
73.60
69.76
59.40
57.11
44.44
43.49
43.00
40.87
33.46
29.10
23.83
20.03
13.82
10.48
f1 (ppm)

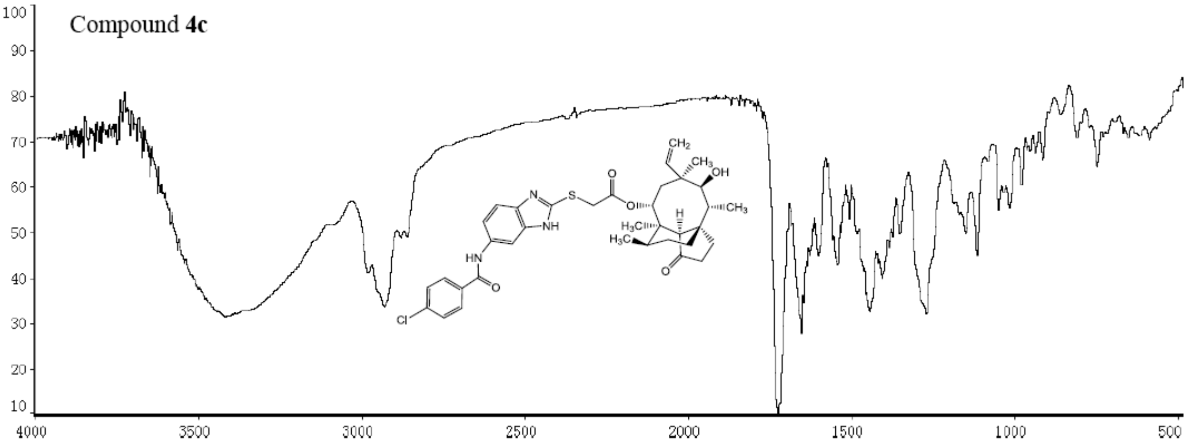
Compound **4c**

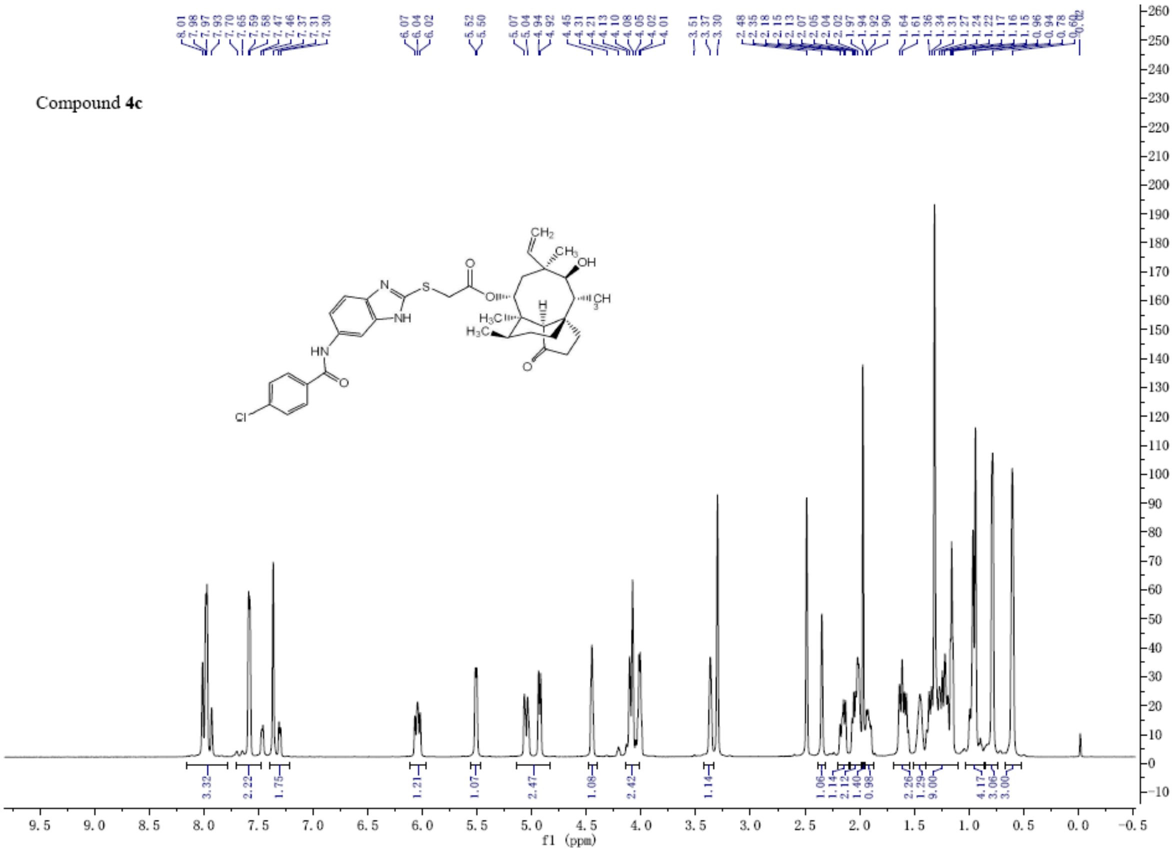
Compound **4c**
f1 (ppm)

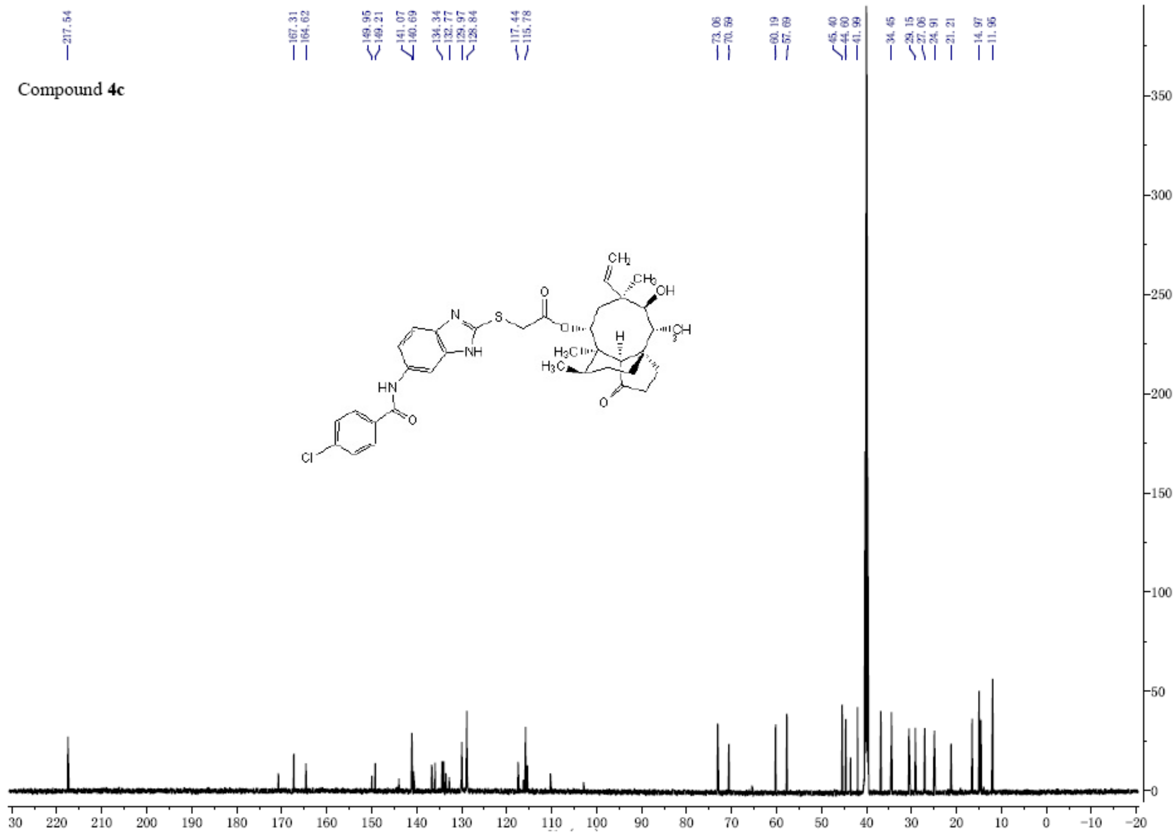
Compound **4c**

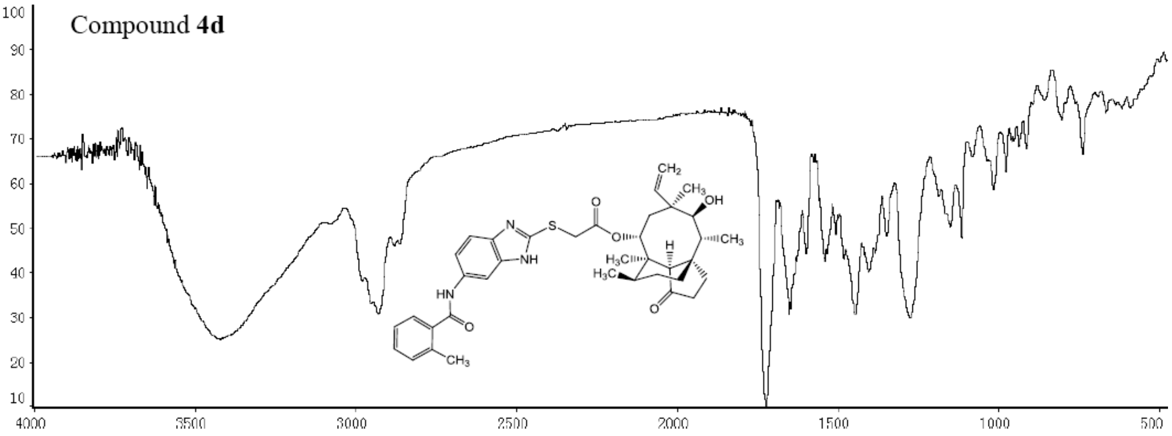
Compound **4d**
100
90
80
70
60
50
40
30
20
10
4000
3500
3000
2500
2000
1500
1000
500
S
O
N
NH
HN
O
CH3
CH2
CH3
OH
CH3
H
H3C
H3C
O

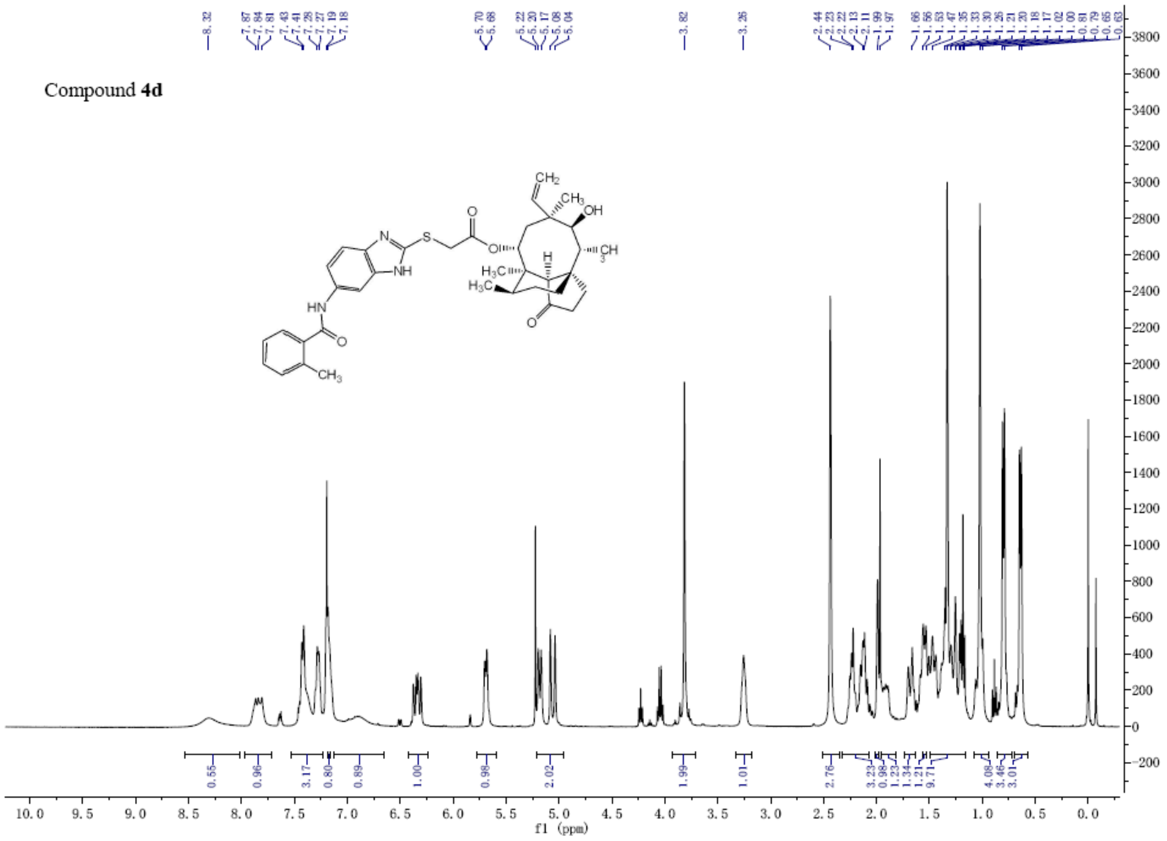
Compound **4d**
f1 (ppm)
10.0
9.5
9.0
8.5
8.0
7.5
7.0
6.5
6.0
5.5
5.0
4.5
4.0
3.5
3.0
2.5
2.0
1.5
1.0
0.5
0.0
0.55
0.96
3.17
0.80
0.89
1.00
0.98
2.02
1.99
1.01
2.76
3.23
0.98
1.34
1.23
1.21
9.71
4.08
3.46
3.01

Compound **4d**

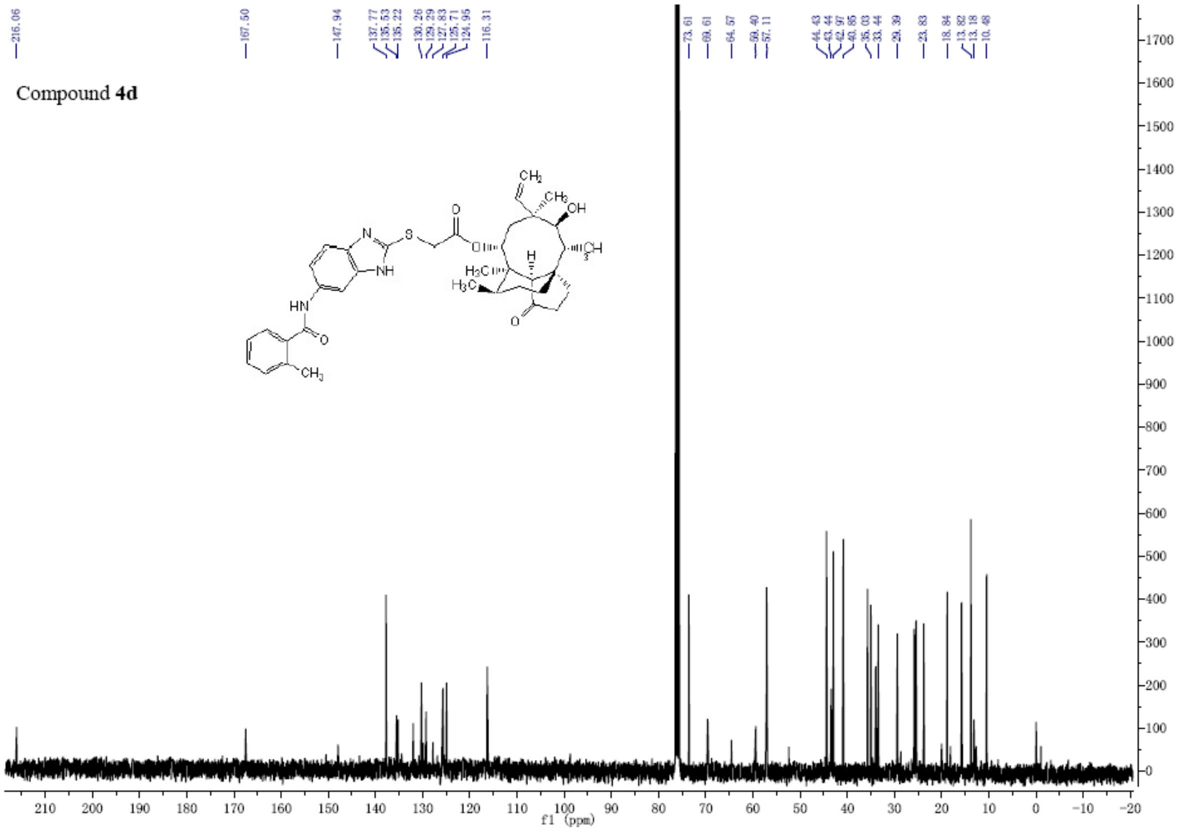

Compound **4e**

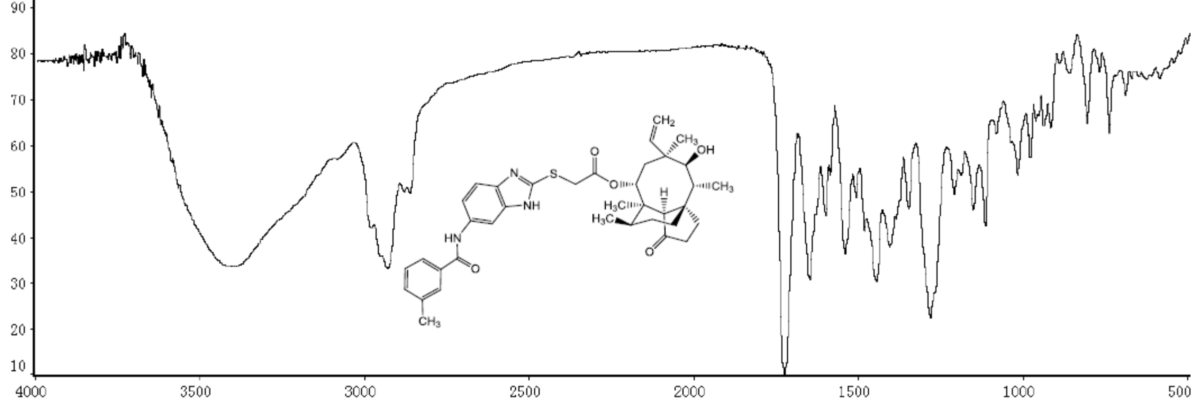

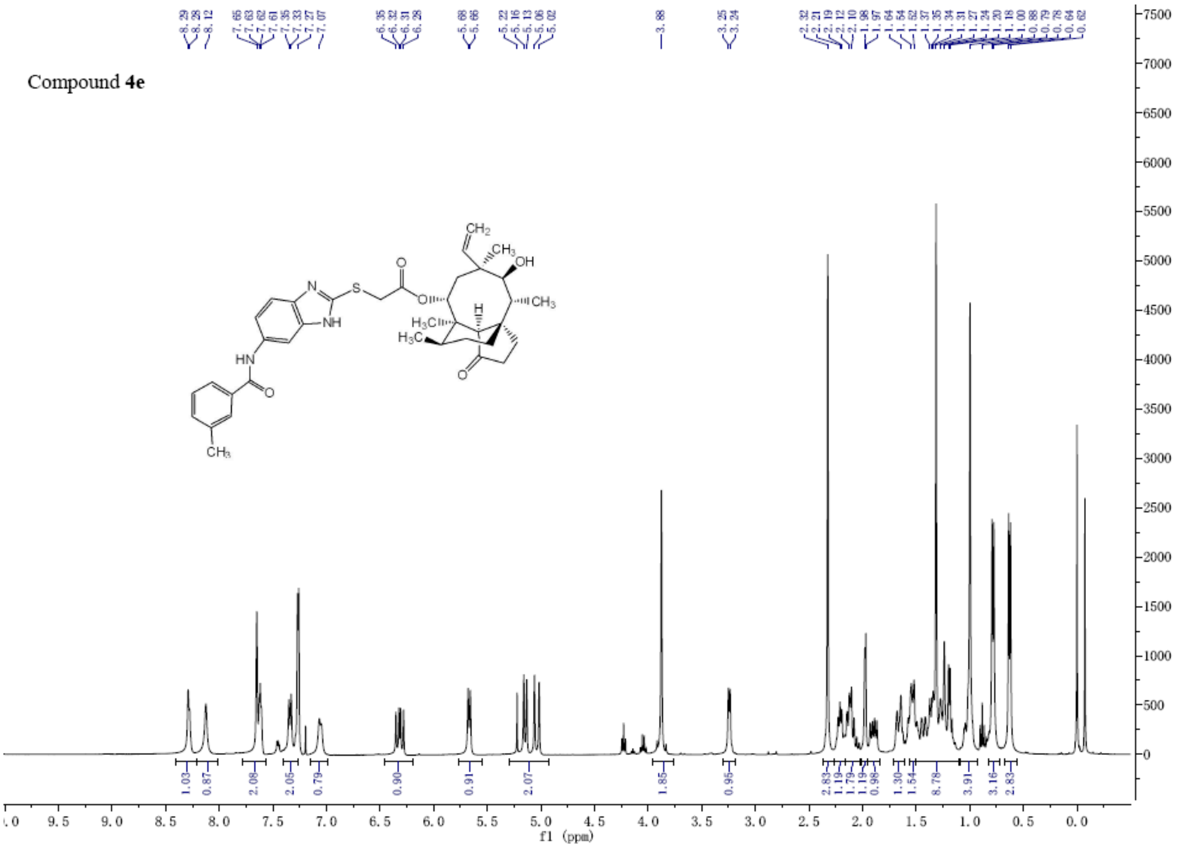
Compound **4e**
f1 (ppm)

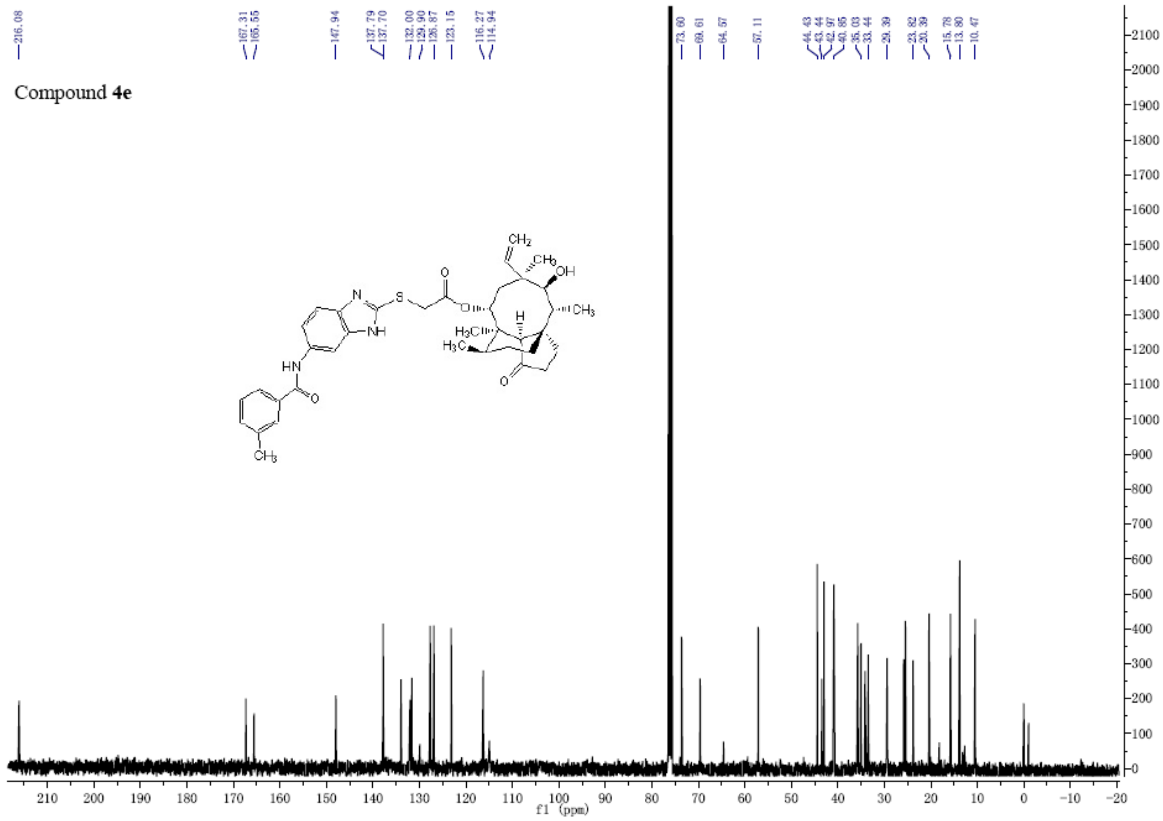
Compound **4e**
f1 (ppm)

Compound **4f**

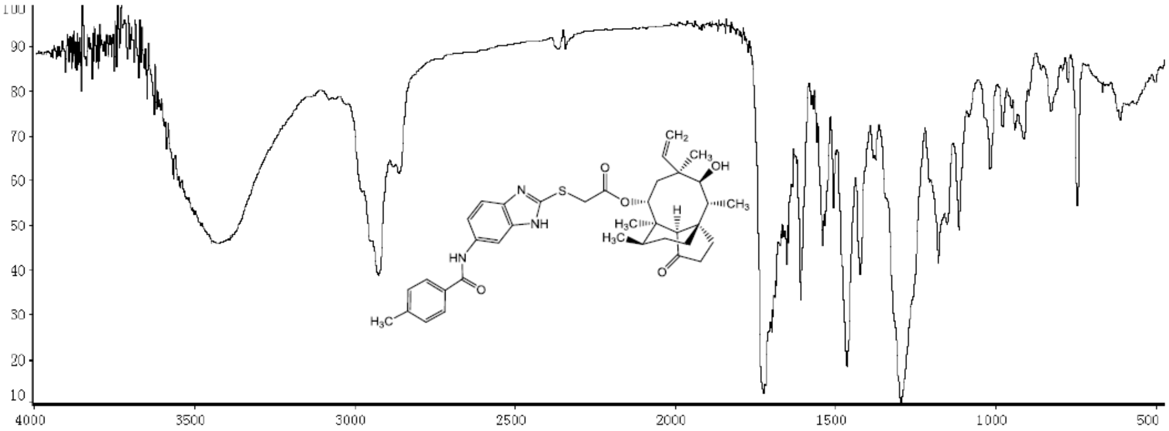

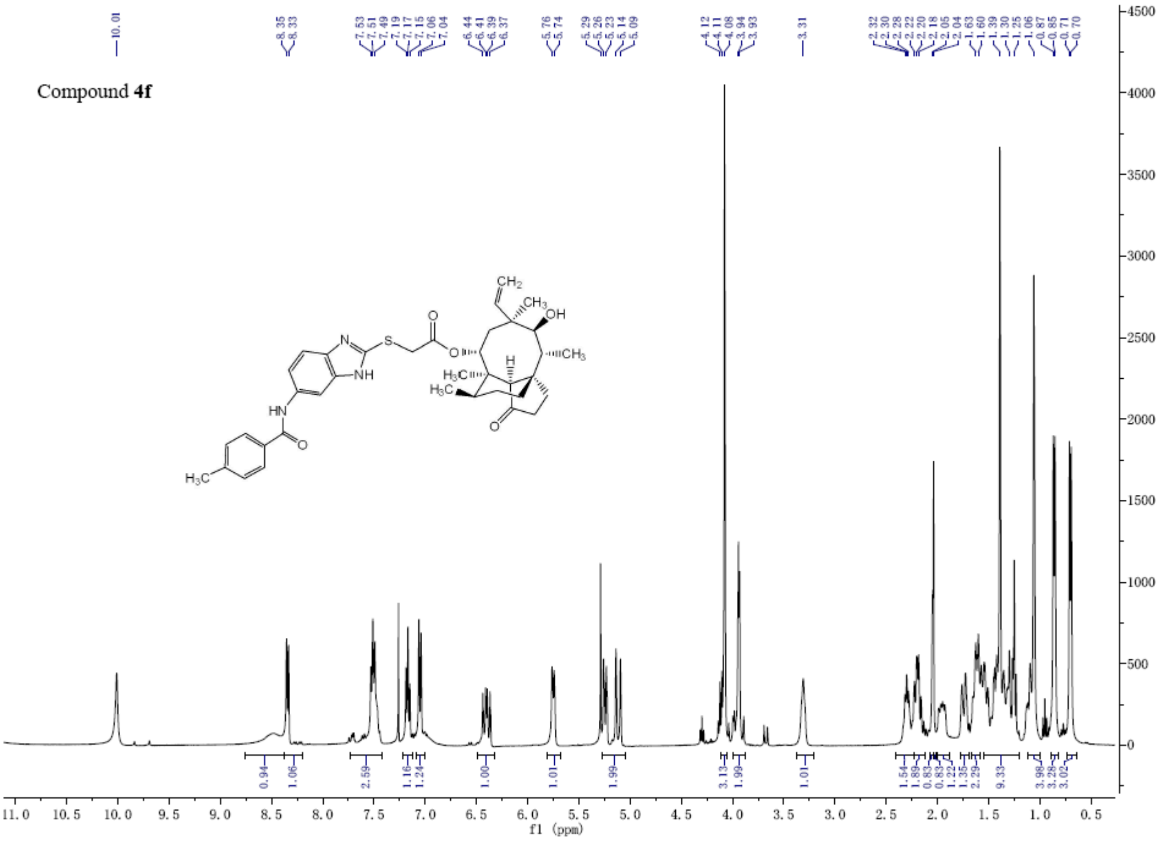

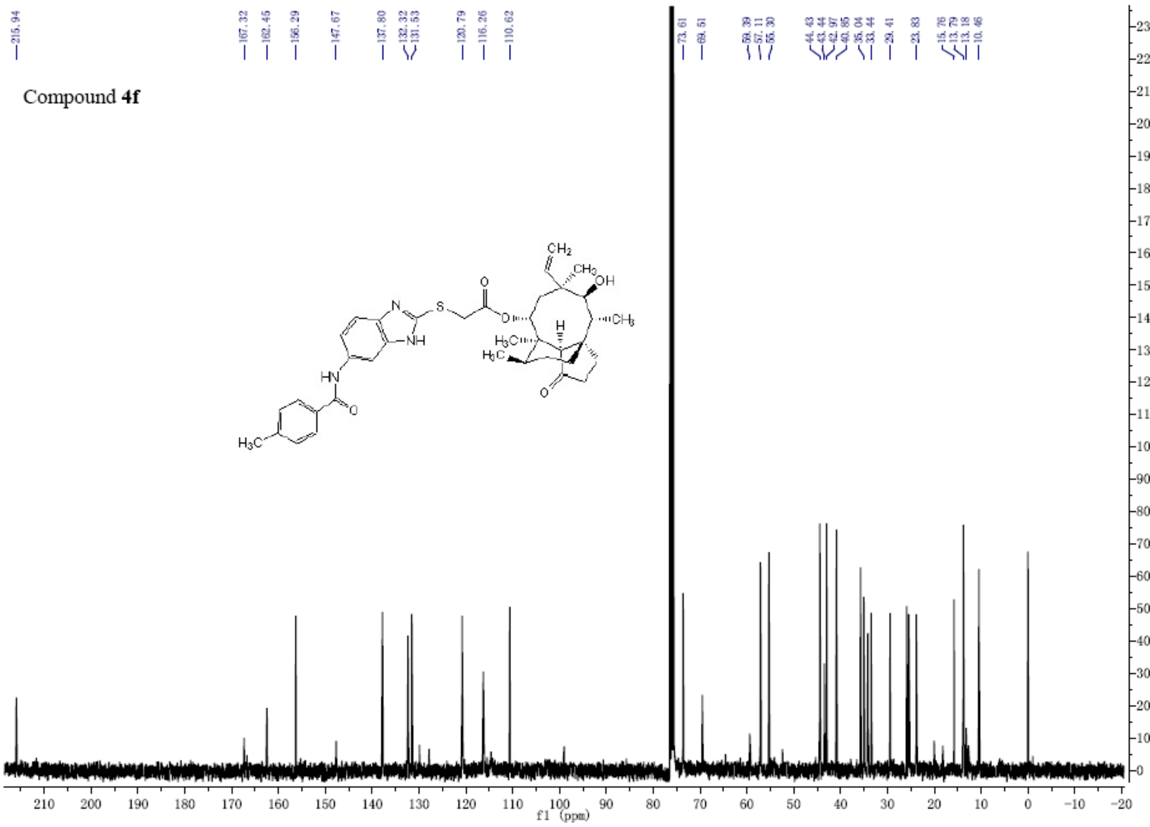
Compound **4f**
215.94
167.32
162.45
156.29
147.67
137.80
132.32
131.53
120.79
116.26
110.62
73.61
69.51
59.39
57.11
55.30
44.43
43.44
42.97
40.85
35.04
33.44
29.41
23.83
15.76
13.79
13.18
10.46
f1 (ppm)

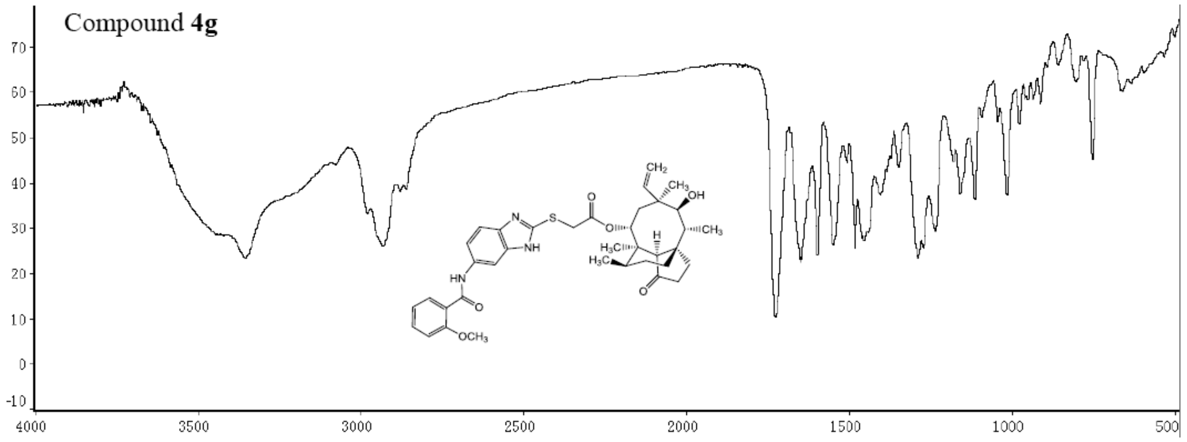
Compound **4g**

Compound **4g**

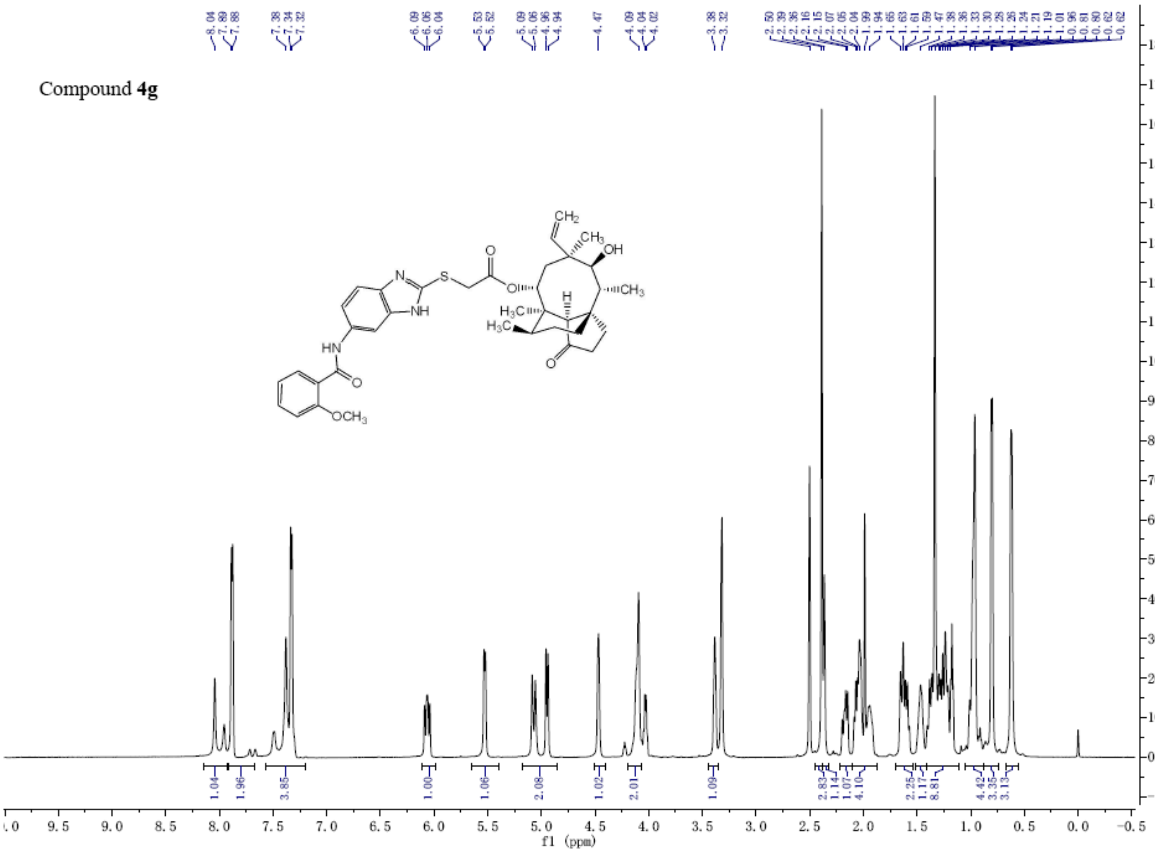

Compound **4g**

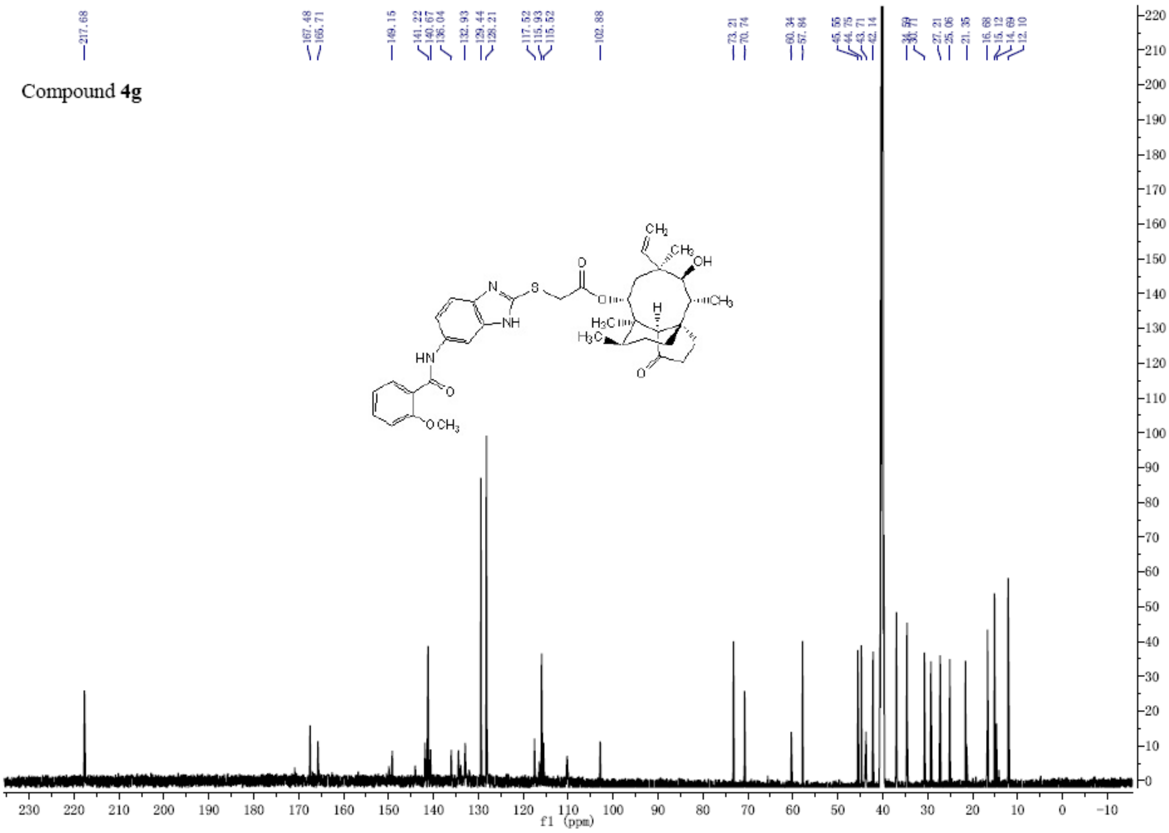

Compound **4h**

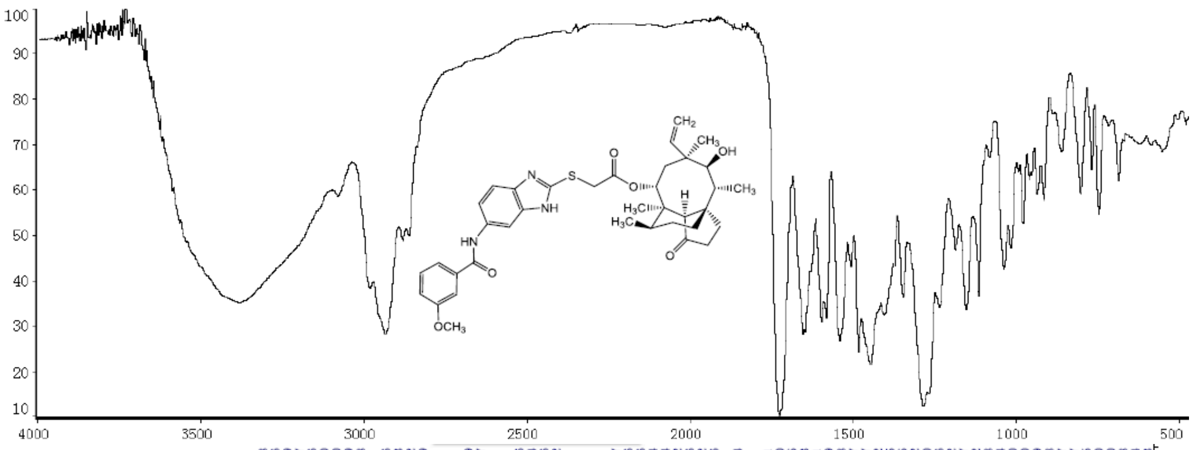

Compound **4h**

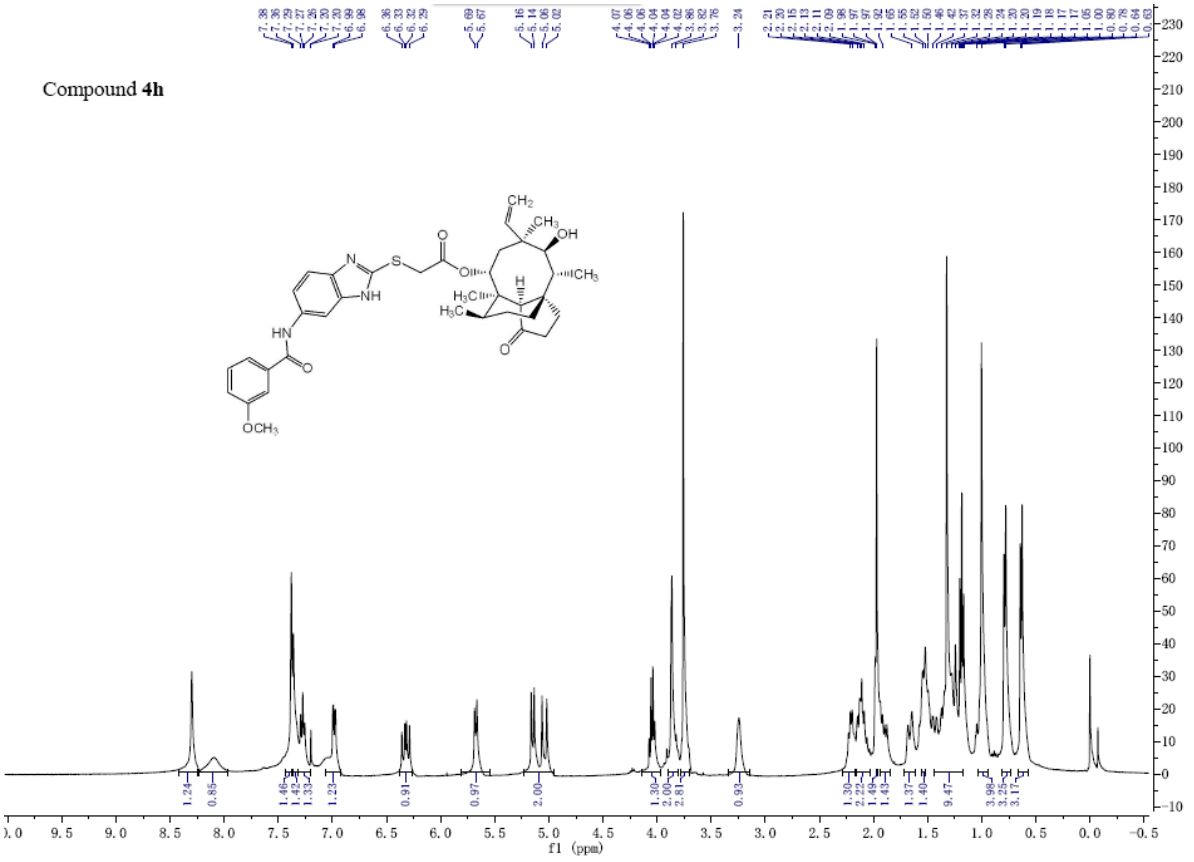

Compound **4h**

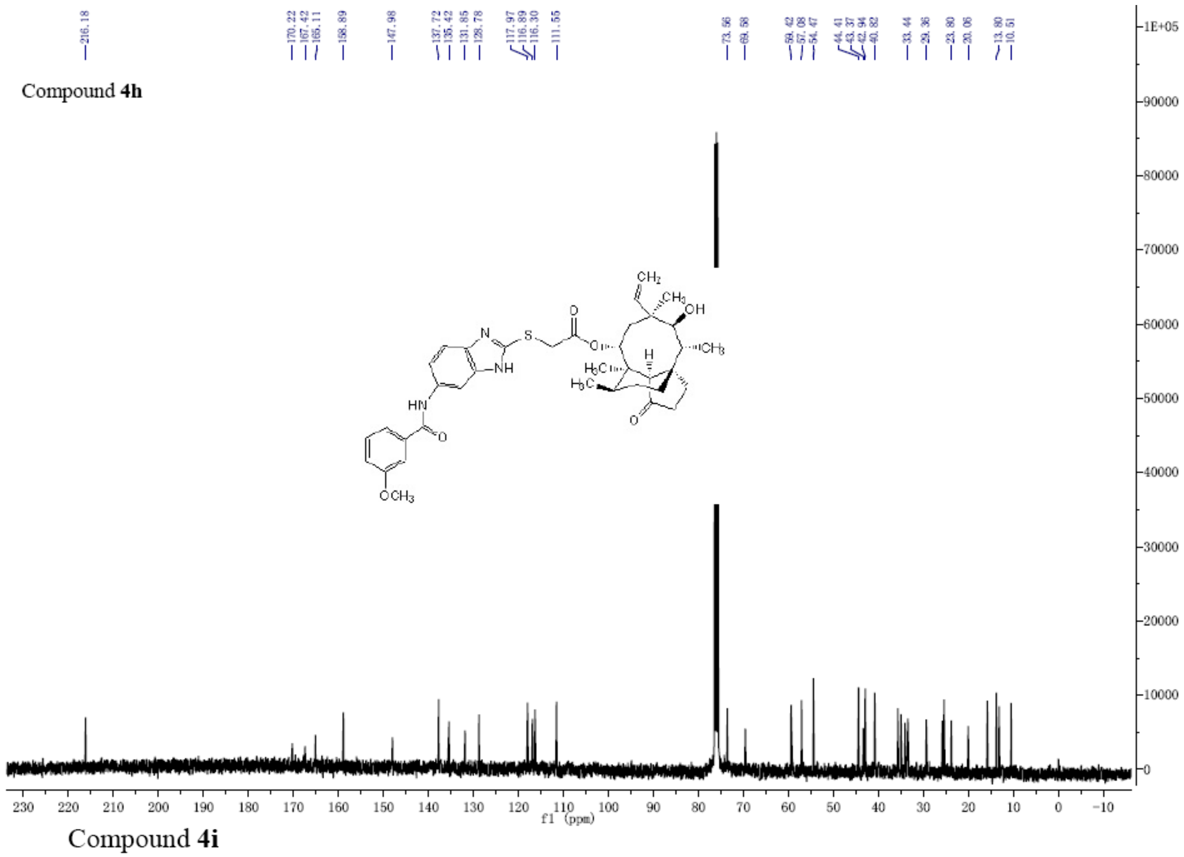

Compound **4i**

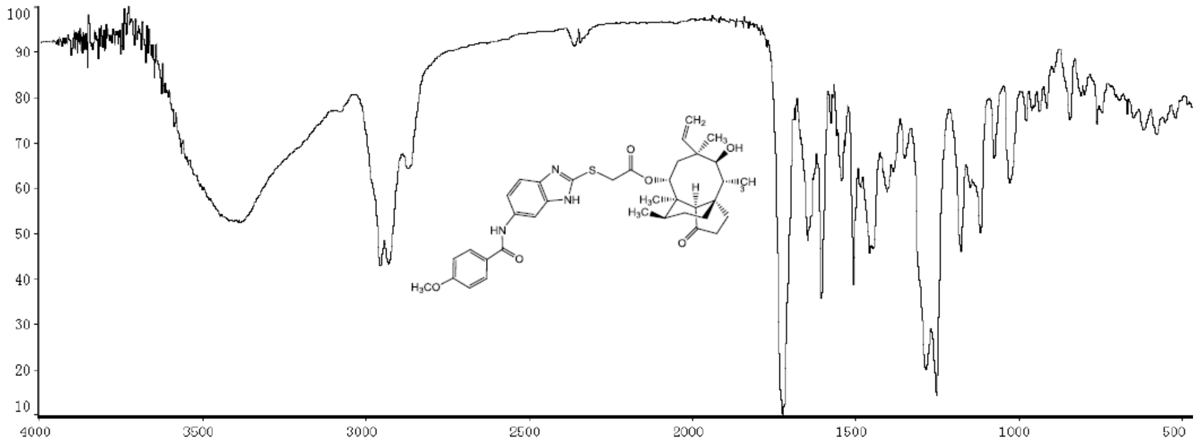

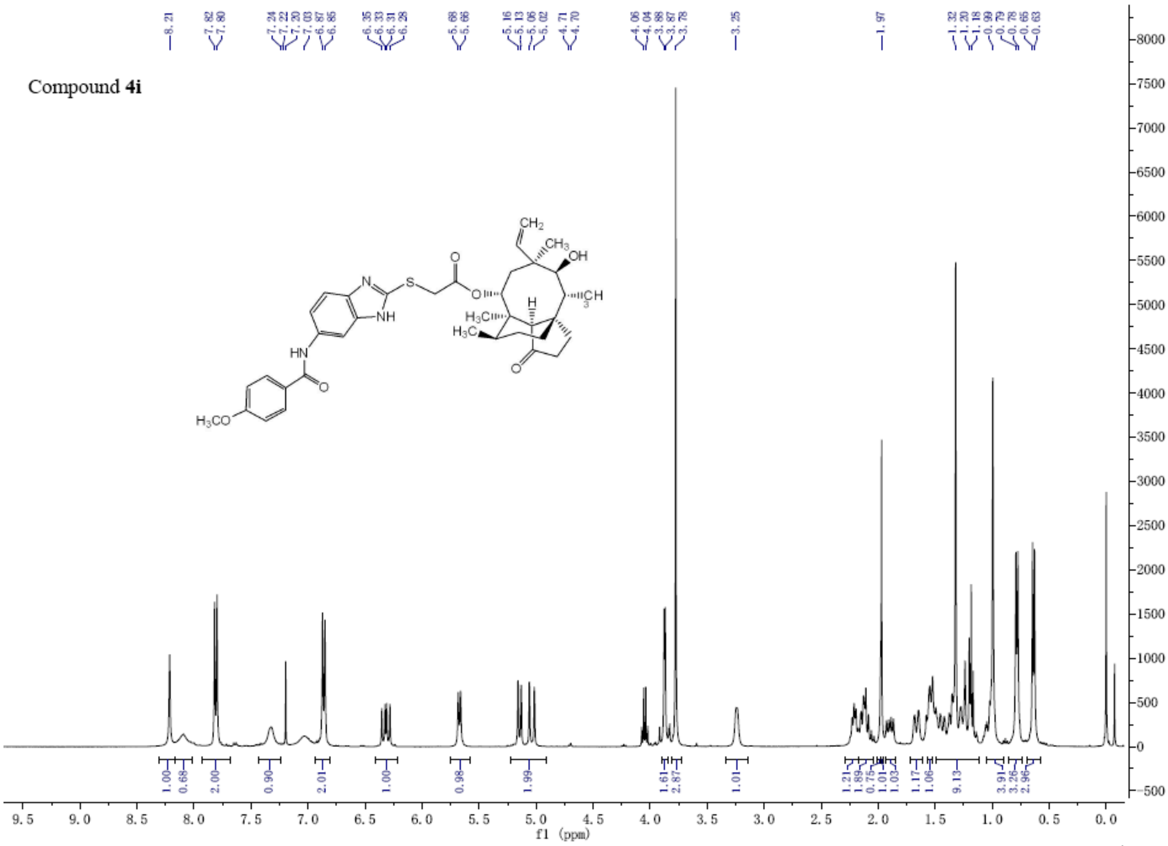
Compound **4i**
8.21
7.82
7.80
7.24
7.22
7.20
7.03
6.87
6.85
6.35
6.33
6.31
6.28
5.68
5.66
5.16
5.13
5.06
5.02
4.71
4.70
4.06
4.04
3.88
3.87
3.78
3.25
1.97
1.32
1.20
1.18
0.99
0.79
0.78
0.65
0.63
1.00
0.68
2.00
0.90
2.01
1.00
0.98
1.99
1.61
2.87
1.01
1.21
1.89
0.75
1.01
1.03
1.17
1.06
9.13
3.91
3.26
2.96
f1 (ppm)

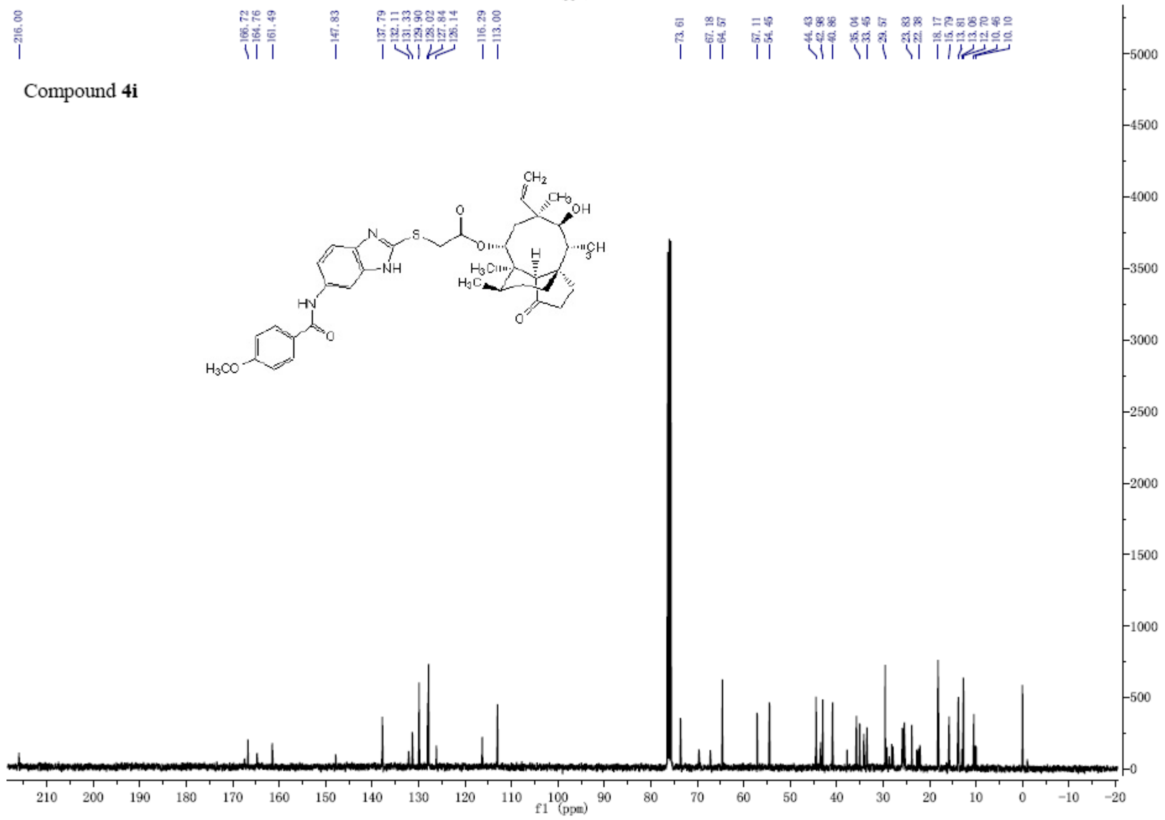
Compound **4i**
216.00
166.72
164.76
161.49
147.83
137.79
132.11
131.33
129.90
128.02
127.84
126.14
116.29
113.00
73.61
67.18
64.57
57.11
54.45
44.43
42.98
40.86
35.04
33.45
29.57
23.83
22.38
18.17
15.79
13.81
13.06
12.70
10.46
10.10
f1 (ppm)

Compound **4j**

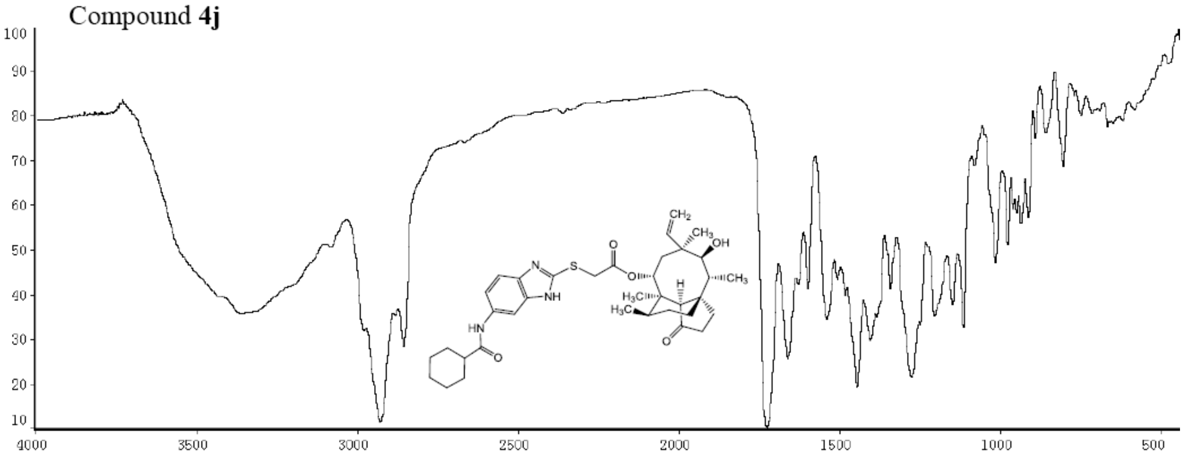

Compound **4j**

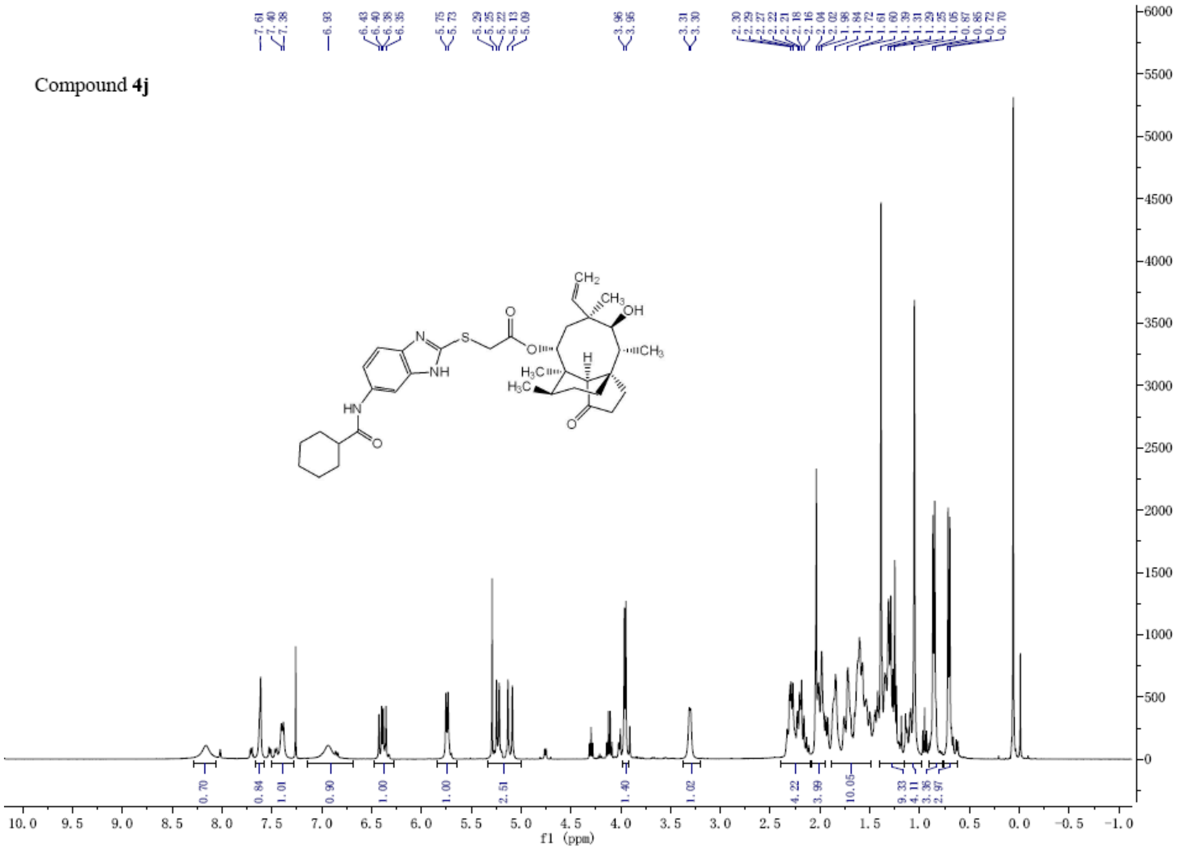

Compound **4j**

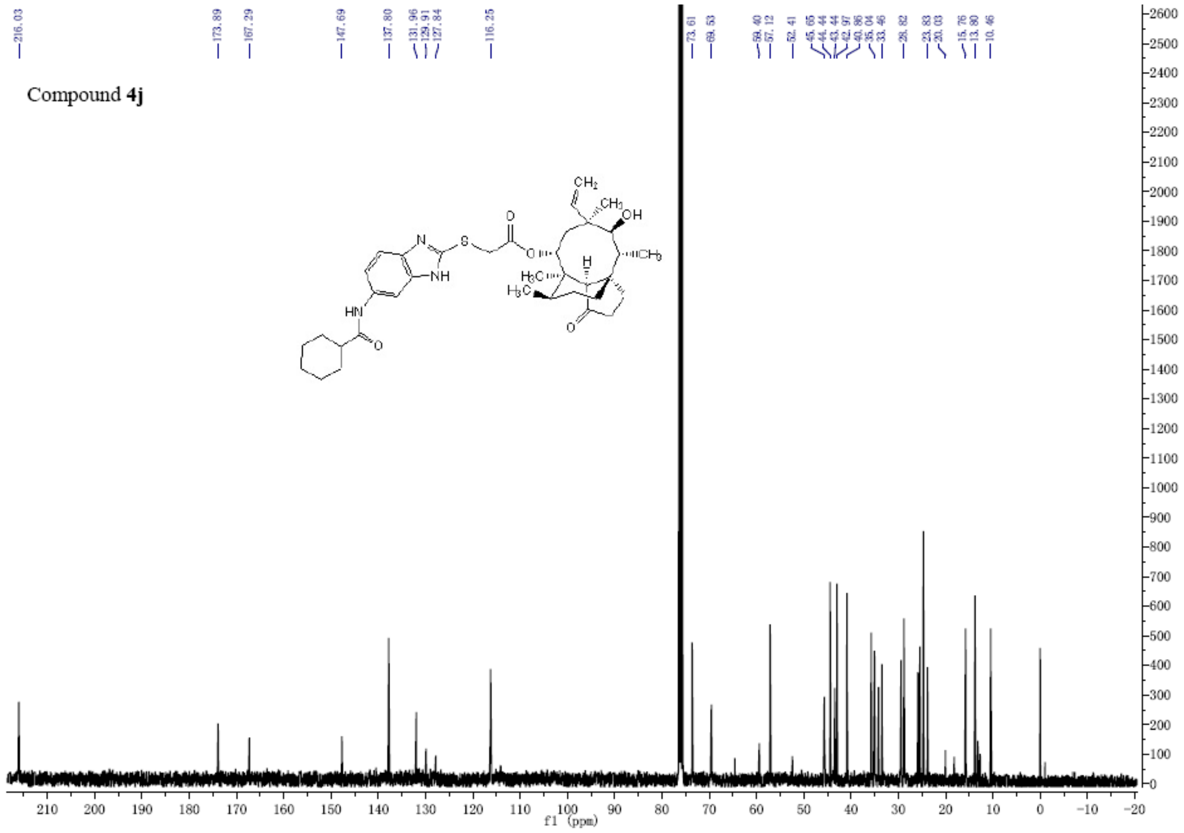

Compound **4k**

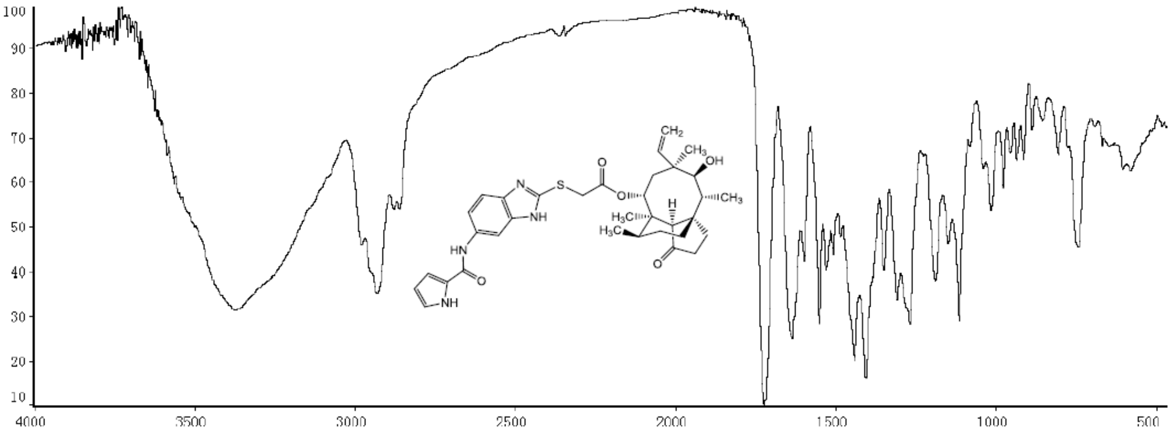

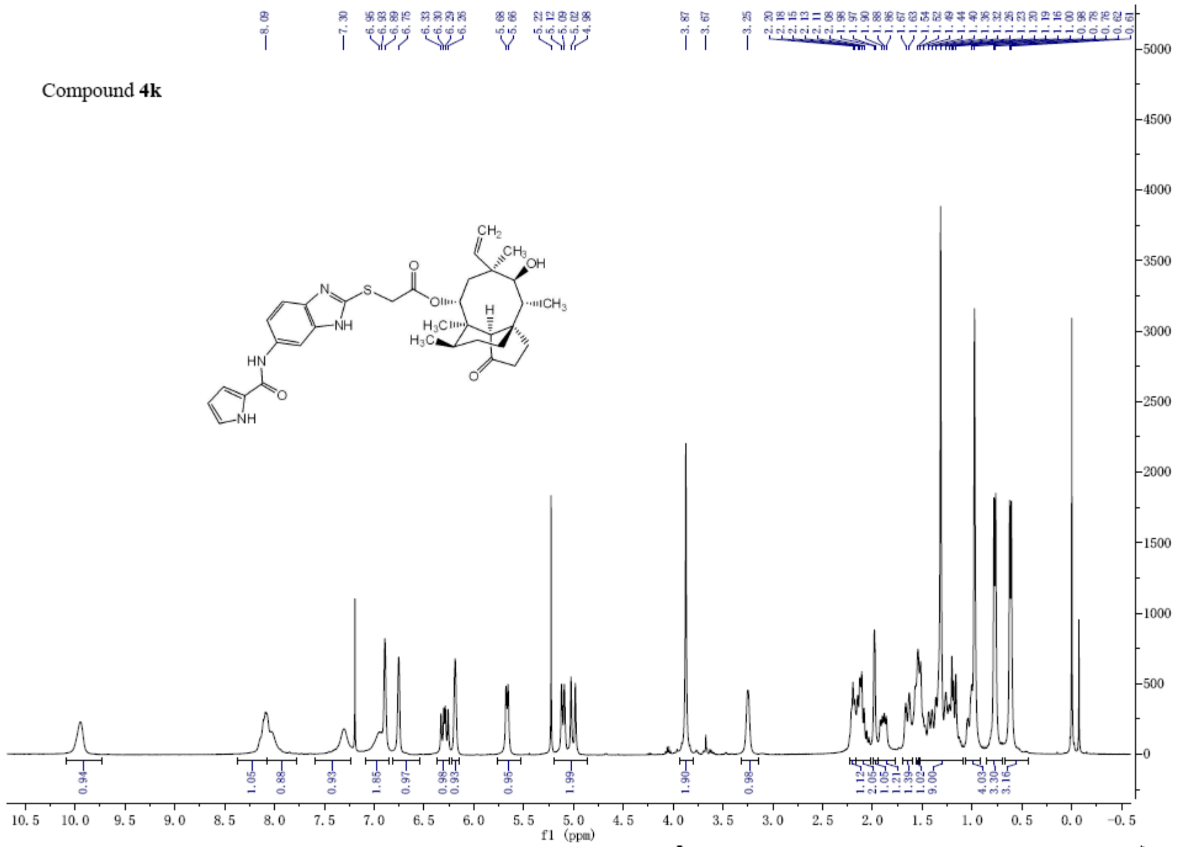
Compound **4k**
f1 (ppm)

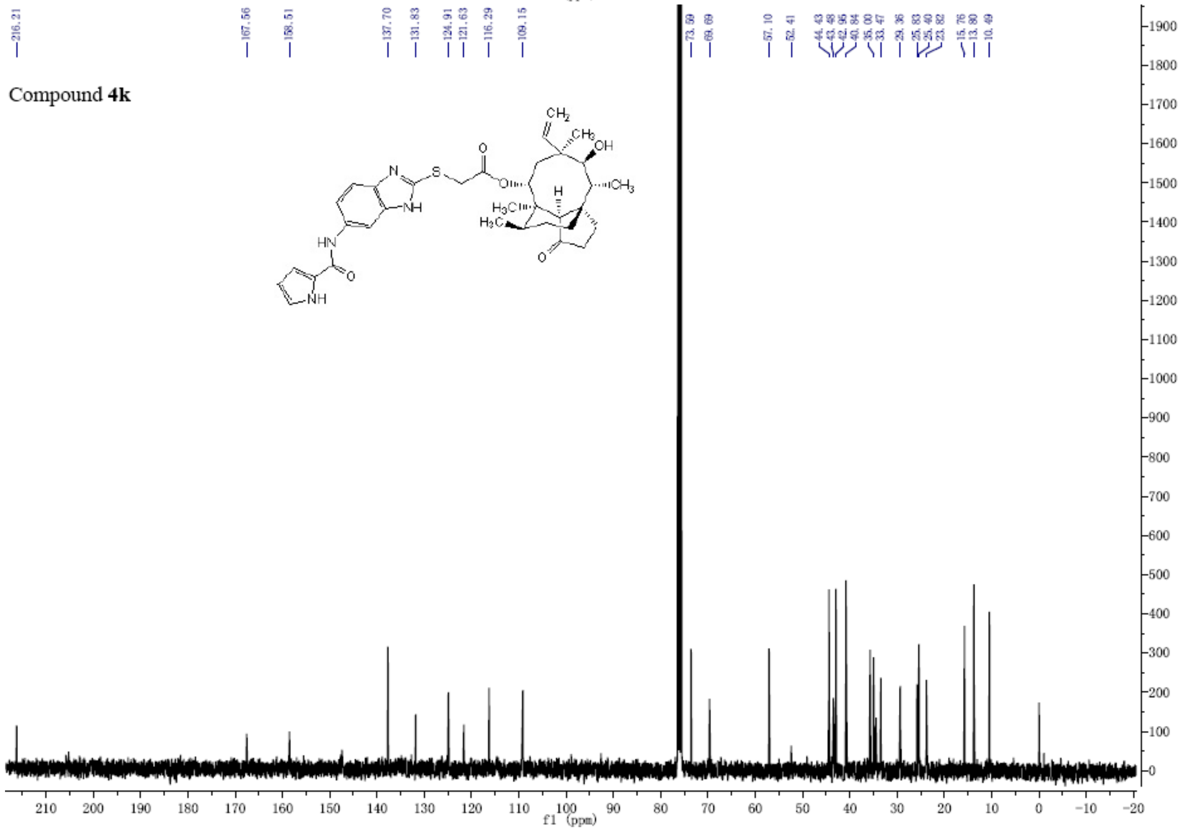
Compound **4k**
f1 (ppm)

Compound **4l**

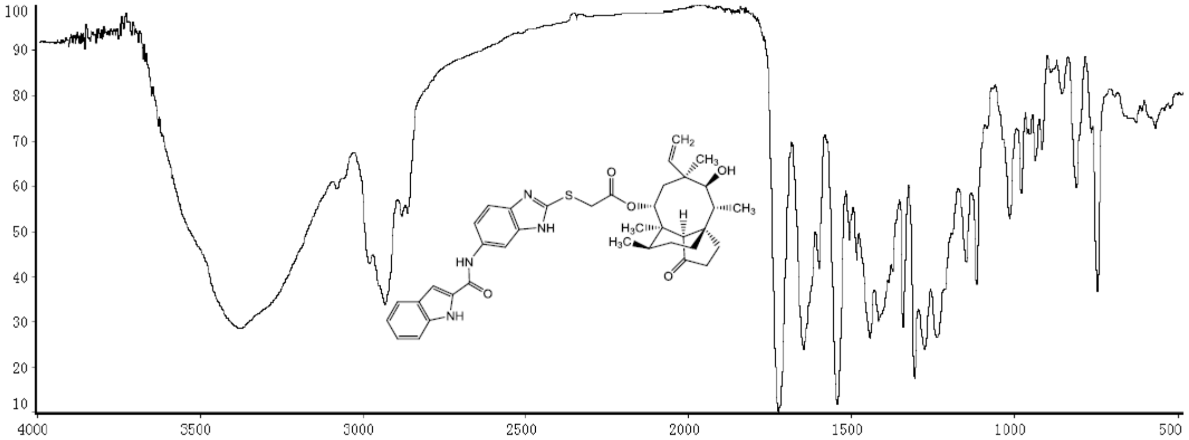

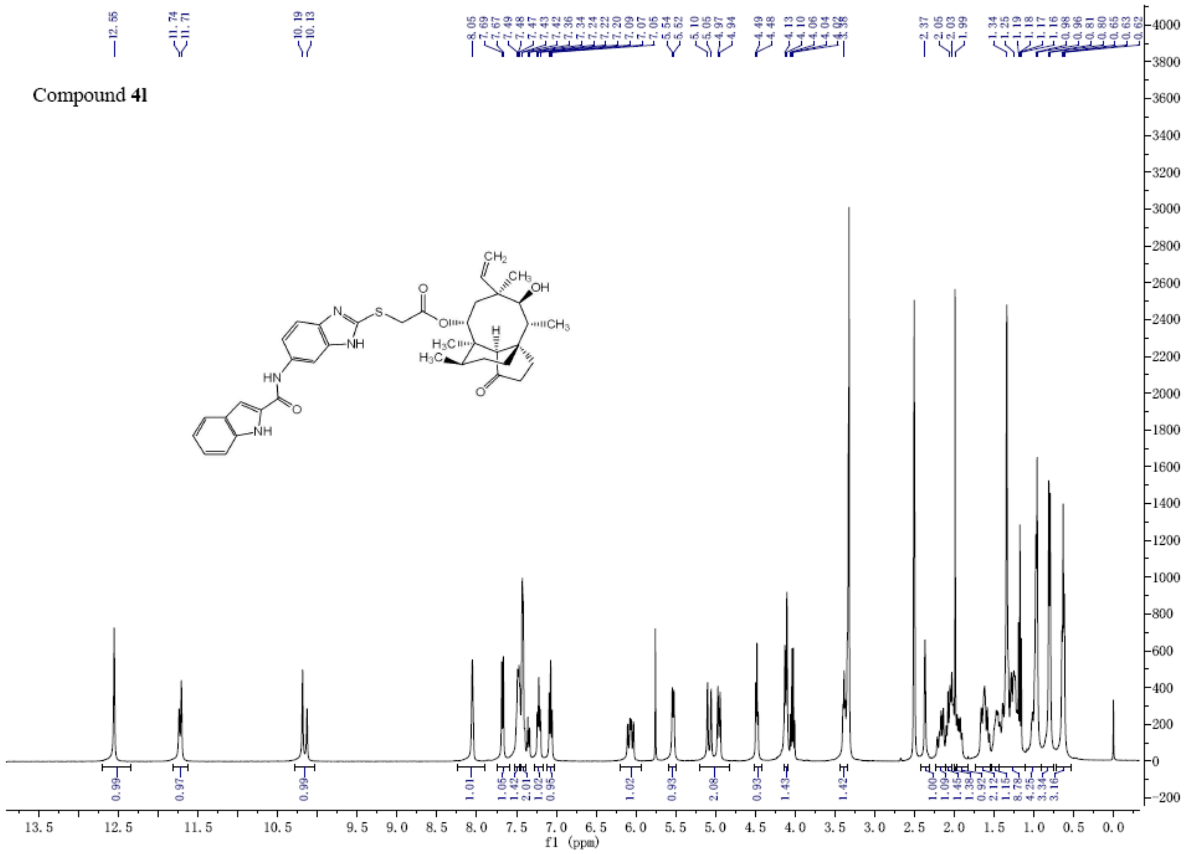

Compound **4l**

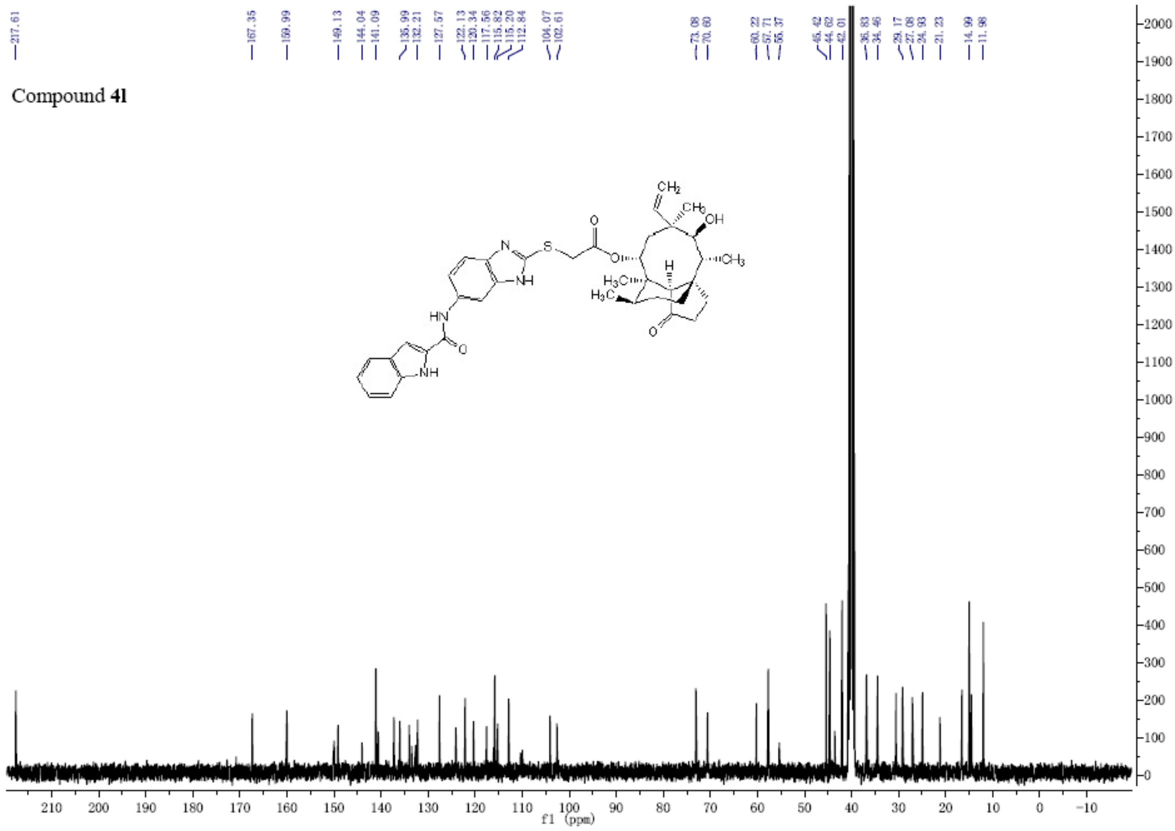

Supplement: Supplementary file 1 [file molecules-21-01488-s001.pdf]
